# Genome rearrangements induce biofilm formation in *Escherichia coli* C – an old model organism with a new application in biofilm research

**DOI:** 10.1186/s12864-019-6165-4

**Published:** 2019-10-22

**Authors:** Jarosław E. Król, Donald C. Hall, Sergey Balashov, Steven Pastor, Justin Sibert, Jennifer McCaffrey, Steven Lang, Rachel L. Ehrlich, Joshua Earl, Joshua C. Mell, Ming Xiao, Garth D. Ehrlich

**Affiliations:** 1 Department of Microbiology & Immunology, Center for Advanced Microbial Processing, Drexel University College of Medicine, 245 N. 15th Street, Philadelphia, PA 19102 USA; 2 Center for Genomic Sciences, Drexel University, Philadelphia, PA USA; 3 Center for Surgical Infections and Biofilms, Institute of Molecular Medicine and Infectious Disease, Drexel University, Philadelphia, PA USA; 4 Department of Microbiology & Immunology, Drexel University, Philadelphia, PA USA; 5 Department of Chemistry, Drexel University, Philadelphia, PA USA; 6 School of Biomedical Engineering, Drexel University, Philadelphia, PA USA; 7 Department of Otolaryngology – Head and Neck Surgery; Drexel University College of Medicine, Drexel University, Philadelphia, PA USA

**Keywords:** *E. coli* biofilm formation, Aggregation, Bacterial stress response, Salt concentration, Temperature stress, Carbon storage regulator, Curli synthesis, Sigma 70, Complete genome sequence

## Abstract

**Background:**

*Escherichia coli* C forms more robust biofilms than other laboratory strains. Biofilm formation and cell aggregation under a high shear force depend on temperature and salt concentrations. It is the last of five *E. coli* strains (C, K12, B, W, Crooks) designated as safe for laboratory purposes whose genome has not been sequenced.

**Results:**

Here we present the complete genomic sequence of this strain in which we utilized both long-read PacBio-based sequencing and high resolution optical mapping to confirm a large inversion in comparison to the other laboratory strains. Notably, DNA sequence comparison revealed the absence of several genes thought to be involved in biofilm formation, including antigen 43, *waaSBOJYZUL* for lipopolysaccharide (LPS) synthesis, and *cpsB* for curli synthesis. The first main difference we identified that likely affects biofilm formation is the presence of an IS3-like insertion sequence in front of the carbon storage regulator *csrA* gene. This insertion is located 86 bp upstream of the *csrA* start codon inside the − 35 region of P4 promoter and blocks the transcription from the sigma^32^ and sigma^70^ promoters P1-P3 located further upstream. The second is the presence of an IS5/IS1182 in front of the *csgD* gene. And finally, *E. coli* C encodes an additional sigma^70^ subunit driven by the same IS3-like insertion sequence. Promoter analyses using GFP gene fusions provided insights into understanding this regulatory pathway in *E. coli*.

**Conclusions:**

Biofilms are crucial for bacterial survival, adaptation, and dissemination in natural, industrial, and medical environments. Most laboratory strains of *E. coli* grown for decades in vitro have evolved and lost their ability to form biofilm, while environmental isolates that can cause infections and diseases are not safe to work with. Here, we show that the historic laboratory strain of *E. coli* C produces a robust biofilm and can be used as a model organism for multicellular bacterial research. Furthermore, we ascertained the full genomic sequence of this classic strain, which provides for a base level of characterization and makes it useful for many biofilm-based applications.

## Background

*Escherichia coli* is a model bacterium and a key organism for laboratory and industrial applications. *E. coli* strain C was isolated at the Lister Institute and deposited into the National Collection of Type Cultures, London, in 1920 (Strain No. 122). It was characterized as more spherical than other *E. coli* strains and its nuclear matter was shown to be peripherally distributed in the cell [1]. *E. coli* C, called a restrictionless strain, is permissive for most coliphages and has been used for such studies since the early 1950’s [2]. Genetic tests showed that *E. coli* C forms an O rough R1-type lipopolysaccharide (LPS), which serves as a receptor for bacteriophages [3]. Its genetic map, which shows similarities to *E. coli* K12, was constructed in 1970 [4]. It is the only *E. coli* strain that can utilize the pentitol sugars, ribitol and D-arabitol, and the genes responsible for those processes were acquired by horizontal gene transfer [5]. Some research on genes involved in biofilm formation in this strain has been attempted but hasn’t been continued (Federica Briani, Università degli Studi di Milano, personal communication) [6].

Biofilm is the most prevalent form of bacterial life in the natural environment [7–11]. However, in laboratory settings, for decades, bacteria have been grown in liquid media in shaking, highly aerated conditions, which select for the planktonic lifestyle. While all laboratory strains of *E. coli*, such as K12, B, W, and Crooks, are poor biofilm formers, environmental isolates usually form robust biofilms. These *E. coli* strains can cause diarrhea and kidney failure, while others cause urinary tract infections, chronic sinusitis, respiratory illness and pneumonia, and other illnesses [12–15]. Many of these symptoms are correlated with biofilms. A few *E. coli* K12 mutant strains have been described as good biofilm formers, such as the *csrA* mutant or AJW678 [16, 17], but one can claim that these mutants cannot occur in natural conditions. Therefore, it is important to find a safe laboratory strain that can serve as a model for biofilm studies.

We found that the *E. coli* C strain forms a robust biofilm under laboratory conditions. The complete genome sequence of this strain was determined and bioinformatics analyses revealed the molecular foundations underlying this phenotype. A combination of experimental and in silico analysis methods allowed us to unravel the two major mechanisms that draw the biofilm formation in this strain.

## Results

### Biofilm formation

**Fig. 1:**
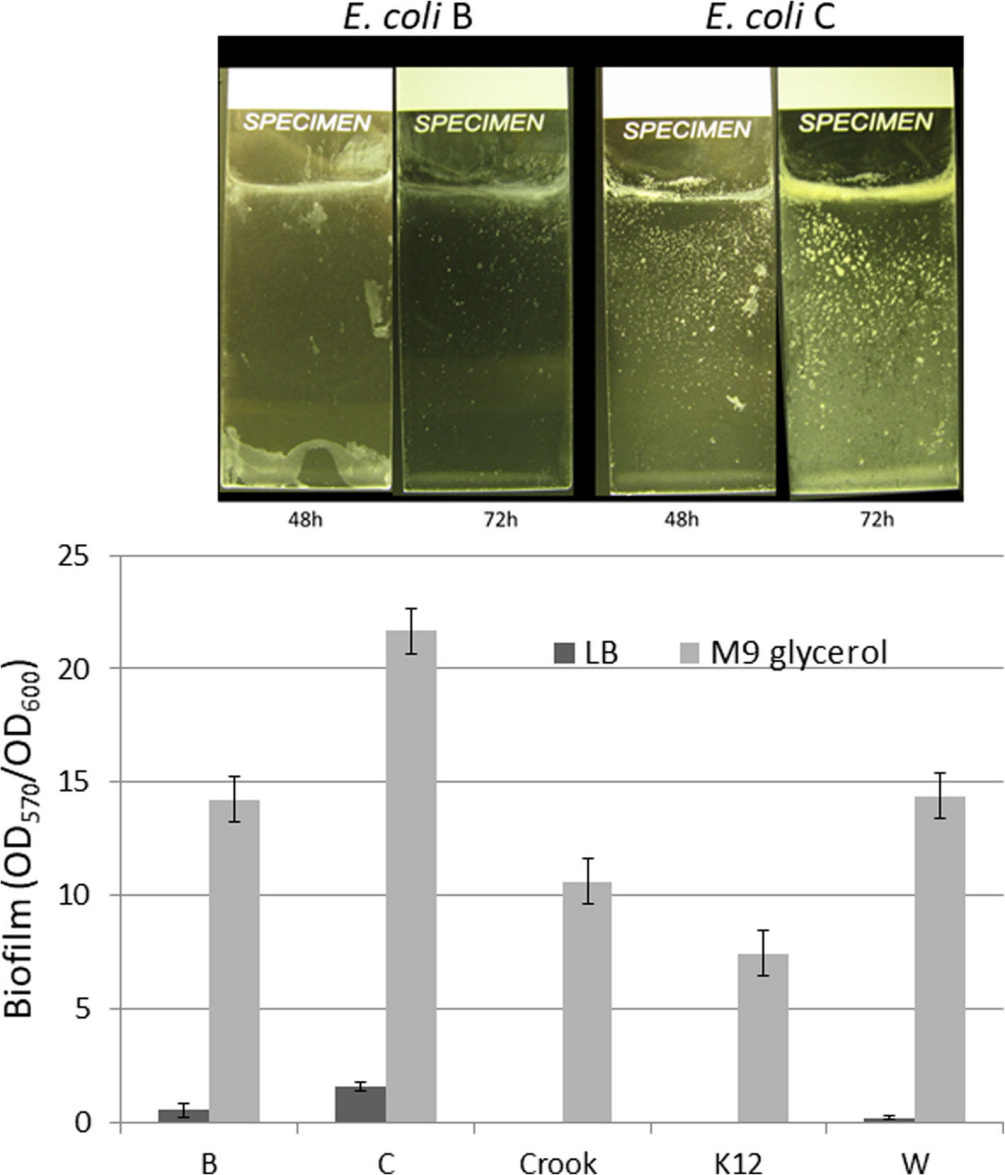
Biofilm formation by *E. coli* strains on (**a**) microscope slides (LB medium) and (**b**) 96-well plates (LB and M9 with glycerol)

In our search for a good model biofilm strain, we screened our laboratory collection of *E. coli* strains using the standard 96-well plate assay [18] and the glass slide assay [19] (Fig. 1a). We found that the *E. coli* C strain formed robust biofilms on both microscope slides and in 96-well plates. In minimal M9 with glycerol medium, the strain C produced 1.5- to 3-fold more biofilm than the other laboratory strains; and in Luria-Bertani (LB) rich medium, the strain C biofilm formation was as much as 7.4-fold higher (Fig. 1b).

**Fig. 2:**
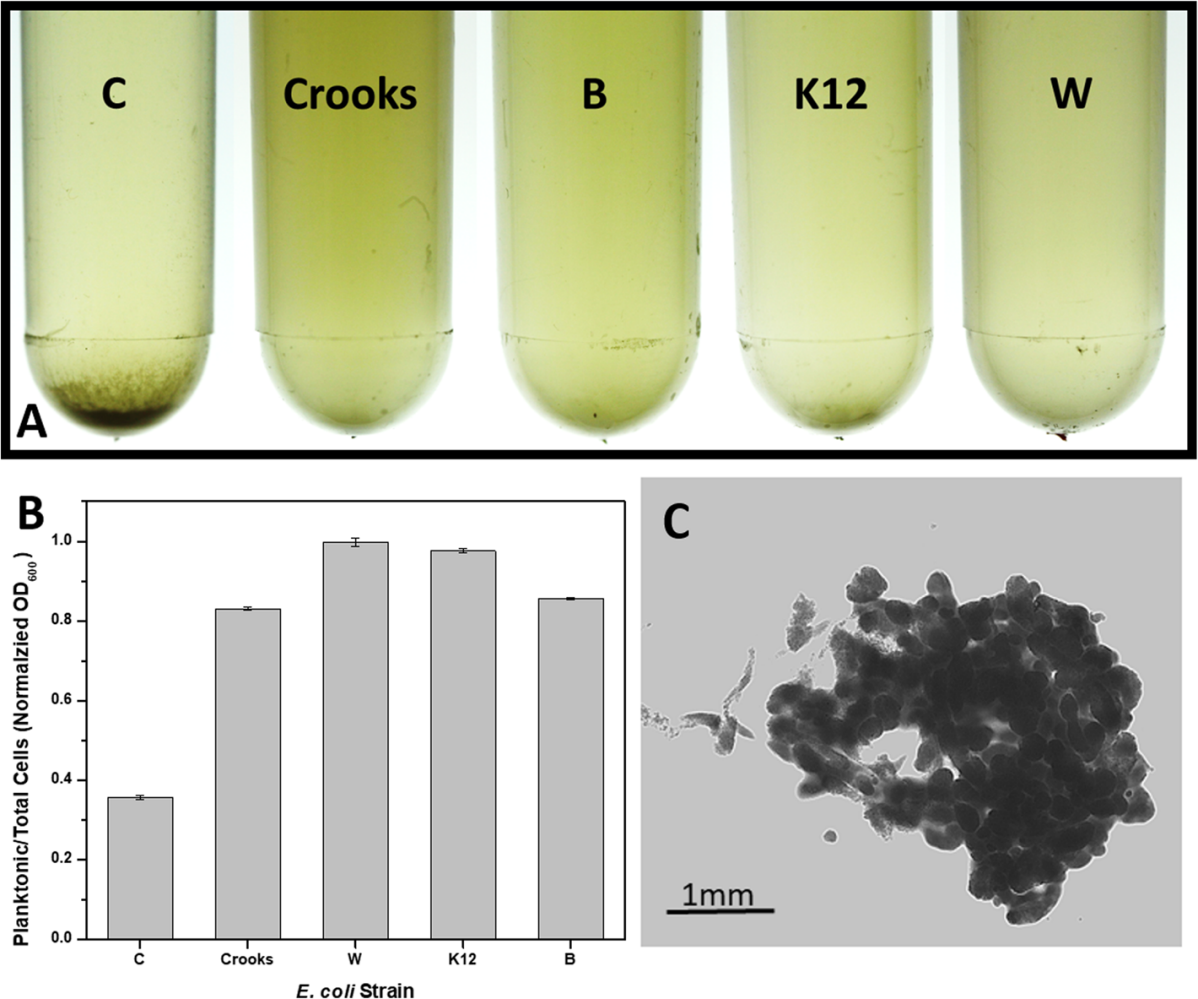
Cell aggregation in overnight culture grown at 30 °C in LB Miller broth on shaker at 250 rpm. **a** From left: *E. coli* C, *E. coli* Crooks, *E. coli* B, *E. coli* K12, and *E. coli* W. **b** Ratio of planktonic cells to total cells measured as OD_600_. **c** Microscopic picture of the *E. coli* C precipitate

During overnight growth in LB medium at 30 °C shaken at 250 rpm, we noticed an increased aggregation of bacterial cells in the *E. coli* C culture (Fig. 2a). The ratio of planktonic cells to total cells in the culture was 0.35 compared to 0.83 and 0.85 for Crooks and B and 0.98 or almost 1 for K12 and W, respectively (Fig. 2b).

### Aggregation at low temperature depends on salt concentration

**Fig. 3:**
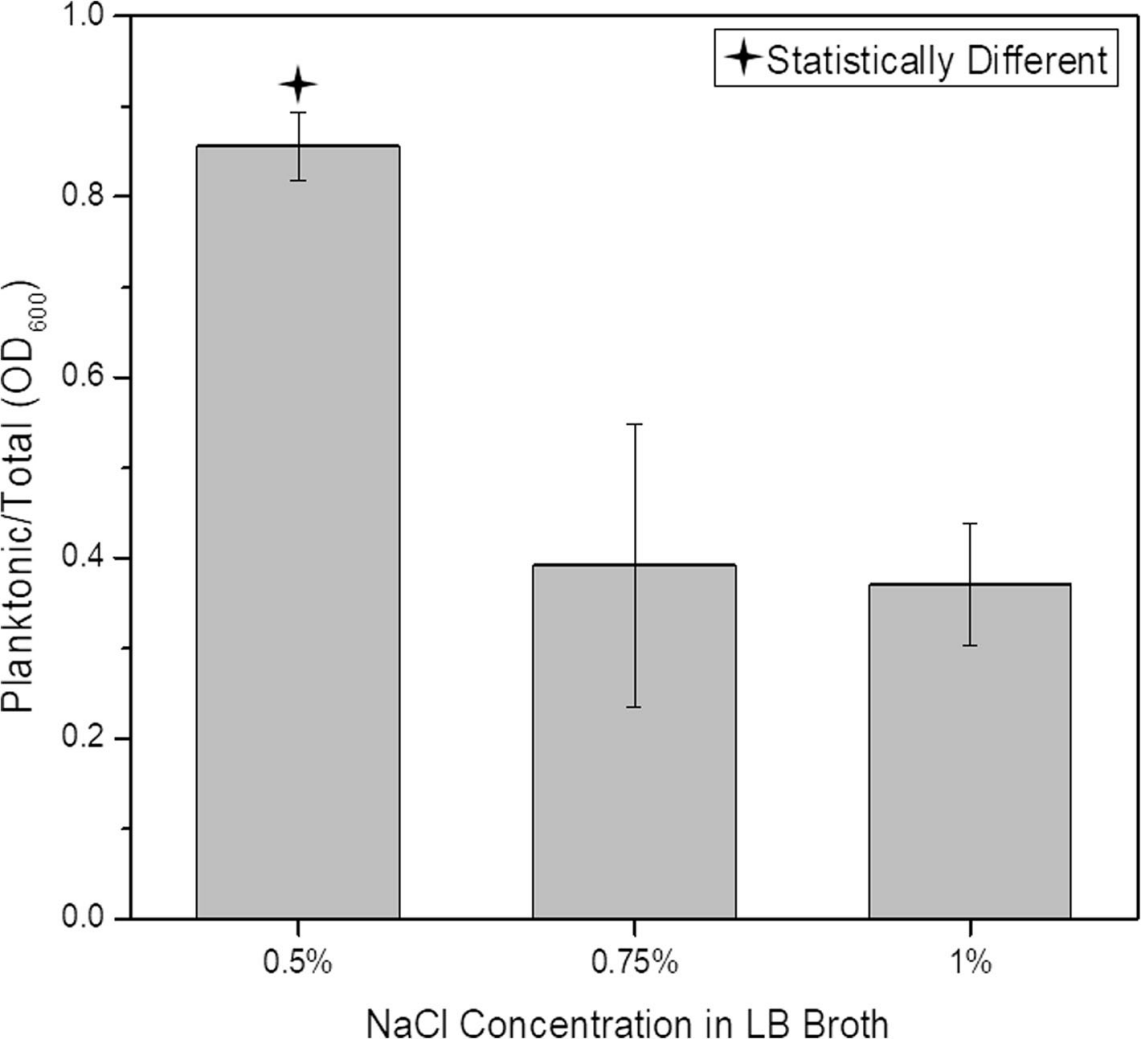
Effect of salt concentration on *E. coli* C aggregation at 30 °C

Previously, we have described a regulatory loop affecting biofilm formation in a high salt/high pH environment. This loop involved the *nhaR*, *sdiA*, *uvrY*, and *hns* genes, as well as the *csrABCD* system [20]. We were interested if the aggregation of *E. coli* C depends on NaCl concentration. We grew the bacteria in three LB broth media containing different amounts of salts: Miller broth (1% NaCl), Lennox broth (0.5% NaCl), and a modified Lennox broth with 0.75% NaCl. After overnight growth at 30 °C in culture tubes shaken at 250 rpm, we observed a lack of aggregation in standard Lennox medium, while in the modified Lennox medium and Miller broth the ratio of planktonic to total cells was similar (Fig. 3). The ratio of planktonic/total cells in the Lennox medium was statistically different (*p* < 0.00001, One-Way ANOVA test) from that in media with a higher NaCl concentration and similar to other strains grown in LB Miller broth (Fig. 2b).

### *E. coli* C genomic sequence

**Fig. 4:**
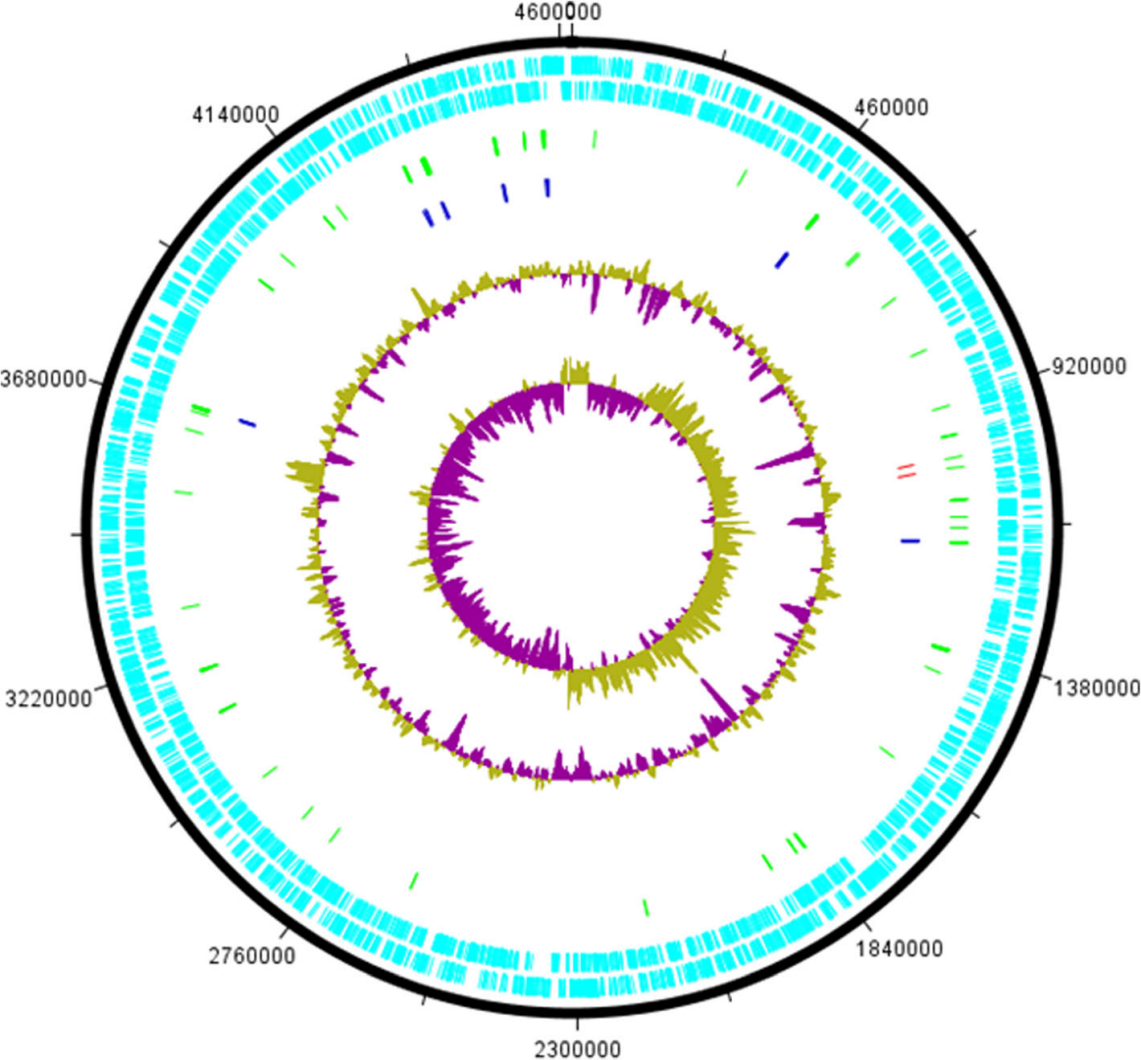
Circular map of the *E. coli* C chromosome (position in bp). The inner circles show GC skew and G + C content. The third circle shows rRNA (blue) and CRISPR (red) clusters. The fourth circle shows hypothetical ORFs (green). Light blue circles represent ORFs on plus and minus strands

The genomes of *E. coli* K12, *E. coli* B, *E. coli* W, and *E. coli* Crooks (GenBank:CP000946) have already been sequenced [21–23]. To compare the genomic sequences of all five laboratory strains, we sequenced the *E. coli* C genome. The chromosome consisted of 4,617,024 bp and encoded 4581 CDSs (Fig. 4). No extrachromosomal DNA was detected. The mean G + C content was 51%. We identified 7 rRNA operons, 89 tRNA genes, and 12 ncRNAs (total 121 RNA genes CP020543.1). The only methylation signal in that genome was Dam methylation. We found that 38,387 out of 38,406 (99.95%) of the GATC motifs had evidence of m6A.

**Fig. 5:**
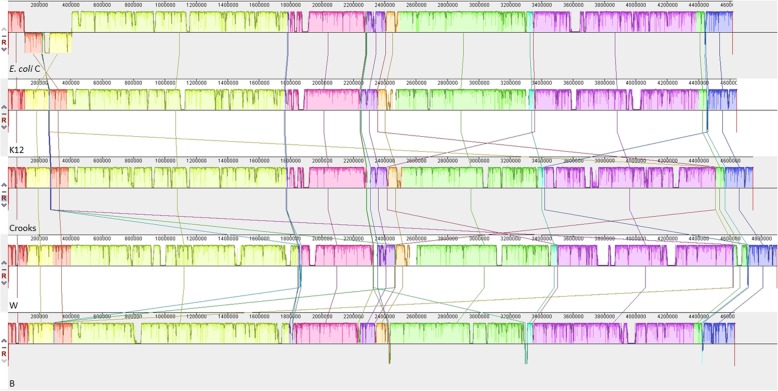
Genome alignment of five *E. coli* strains using Mauve. Each chromosome has been laid out horizontally and homologous blocks in each genome are shown as identically colored regions linked across genomes. The inverted region in *E. coli* C is shifted below the genome’s center axis. From the top: *E. coli* C, K12, Crooks, W, and B

**Fig. 6:**
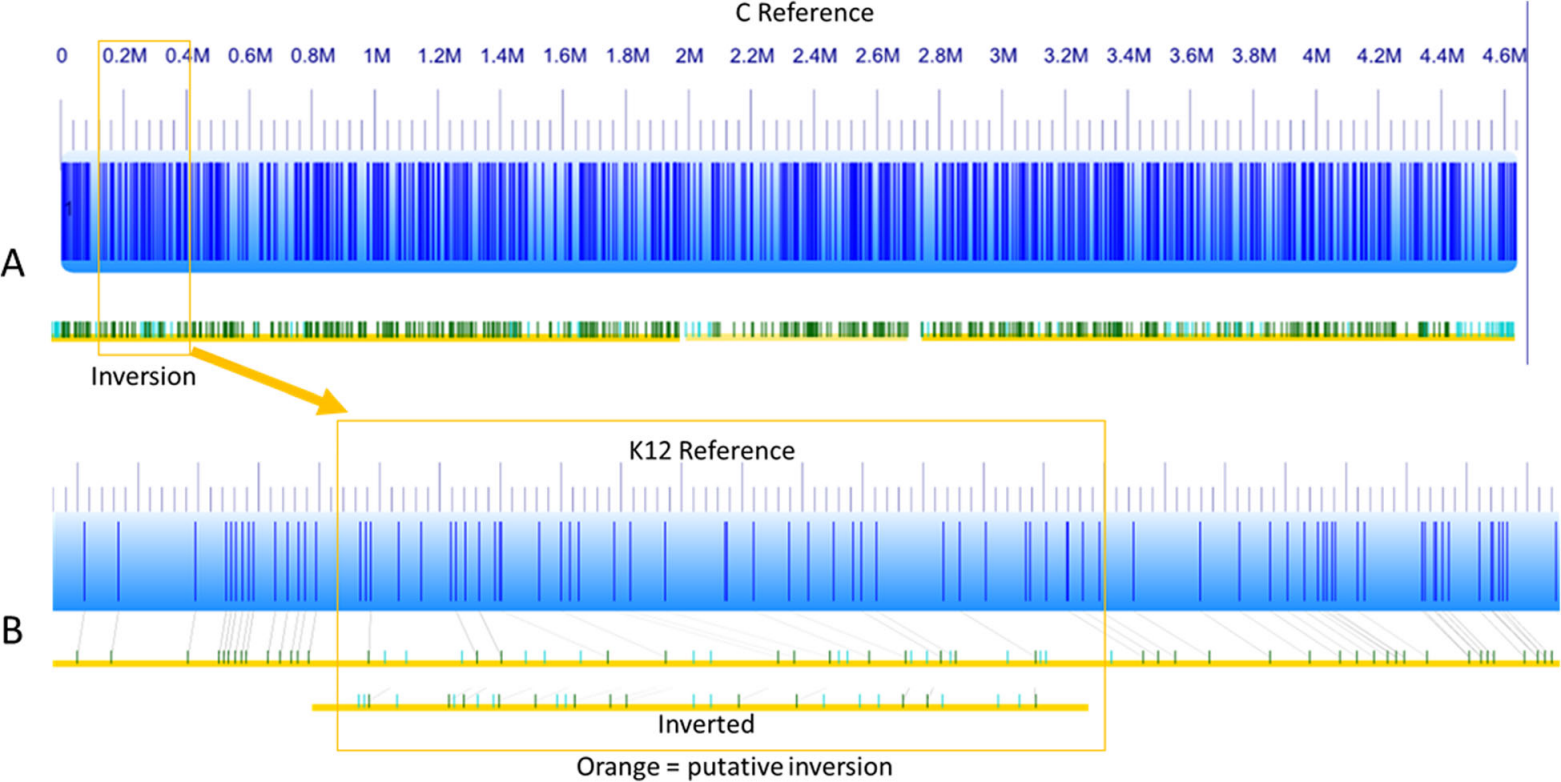
Optical mapping of *E. coli* C chromosome and comparison to K12 strain. **a** In silico generated map (blue) and optical map (yellow/green) of *E. coli* C. **b** In silico generated map (blue) and optical map (yellow/green) of *E. coli* K12

Comparison with other laboratory *E. coli* strains showed a high degree of synteny except for an inverted 300 kb region between 107 and 407 kb (Fig. 5). That inverted region showed also an inverted GC skew in comparizon to the flanking regions, indicating a recent inversion event or an assembly error (Fig. 4). To prove that the inversion represented an actual event, we used an optical mapping method [24]. The order of obtained fluorescently labelled fragments was identical with the in silico constructed map of the *E. coli* C chromosome (Fig. 6a), indicating the authenticity of the inversion.

A similar comparison of *E. coli* K12 maps confirmed the stringency and precision of the optical mapping results (Fig. 6b). Comparison between the two optical maps confirmed that the PacBio-predicted inversion of the 300-kb DNA fragment was indeed a real event (Fig. 6b).

### Genetic content

**Fig. 7:**
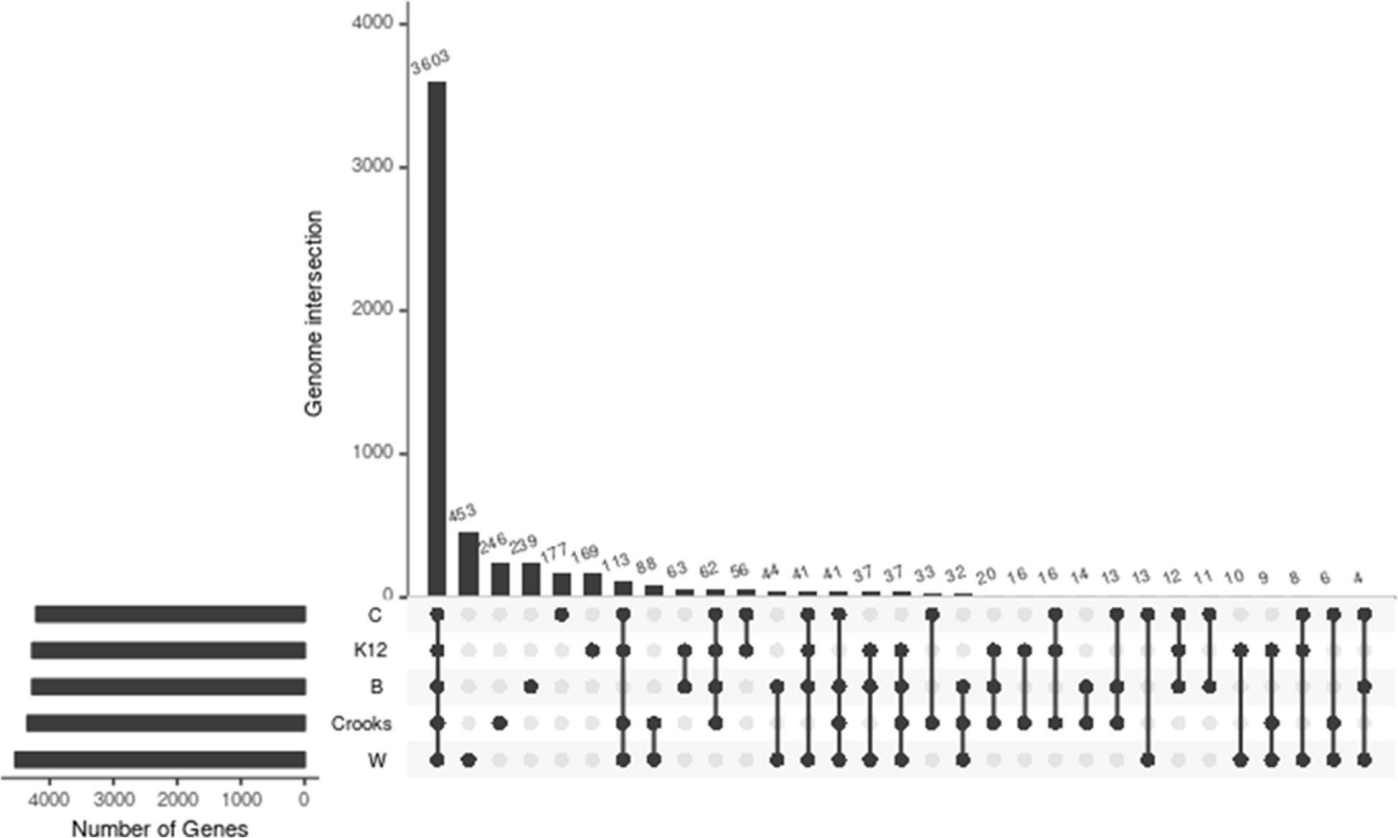
Comparison of orthologous CDSs among C, W, K-12, B, and Crooks strains. The number of shared genes, the number (log_10_) of unique genes, and the genes shared between one, two, three, and four strains are shown. Graph was generated with the UpSet software [25]

A maximal likelihood tree showed that *E. coli* C was most similar to the K12 strain (Additional file 1: Figure S1). A comparison of chromosomal protein-coding orthologs among the laboratory strains showed that, out of the 5686 predicted CDSs, 3603 were shared among all five strains. Only 37 genes were present in all four of the other lab strains that were absent in *E. coli* C (Additional file 11: Table S1) (Fig. 7). Out of 177 genes that were unique to *E. coli C,* 108 encoded transposases or unknown proteins and 69 CDSs showed homology to known proteins (Table 1).

**Table 1:** Sixty-nine unique genes in *E. coli* C genome

Lp	Gene Name or Prokka group	Function - gene description
1	mhpB_2	2,3-Dihydroxyphenylpropionate/2,3-dihydroxicinnamic acid 1,2-dioxygenase
2	yniC_1	2-Deoxyglucose-6-phosphate phosphatase
3	mhpC_2	2-Hydroxy-6-oxononadienedioate/2-hydroxy-6-oxononatrienedioate hydrolase
4	mhpD_2	2-Keto-4-pentenoate hydratase
5	mhpA_2	3-(3-Hydroxy-phenyl)propionate/3-hydroxycinnamic acid hydroxylase
6	mhpE_2	4-Hydroxy-2-oxovalerate aldolase
7	pcaK_2	4-Hydroxybenzoate transporter PcaK
8	mhpF_2	Acetaldehyde dehydrogenase
9	group_2290	Acetyltransferase (GNAT) family protein
10	group_2332	Alpha/beta hydrolase family protein
11	csbX_1	Alpha-ketoglutarate permease
12	csbX_2	Alpha-ketoglutarate permease
13	group_2293	Ankyrin repeats (3 copies)
14	group_2295	Ankyrin repeats (3 copies)
15	group_2370	Ankyrin repeats (3 copies)
16	clpC	ATP-dependent Clp protease ATP-binding subunit ClpC
17	ftsH3	ATP-dependent zinc metalloprotease FtsH 3
18	ftsH4	ATP-dependent zinc metalloprotease FtsH 4
19	group_2325	Cell envelope integrity inner membrane protein TolA
20	bcsA_2	Cellulose synthase catalytic subunit [UDP-forming]
21	wzzB_1	Chain length determinant protein
22	group_2304	Colanic acid exporter
23	dtpD_2	Dipeptide permease D
24	group_337	DNA-binding transcriptional regulator AraC
25	group_258	Esterase YqiA
26	group_343	Fructosamine kinase
27	group_2307	GalNAc(5)-diNAcBac-PP-undecaprenol beta-1,3-glucosyltransferase
28	group_2365	Glutathione-regulated potassium-efflux system protein KefC
29	ltrA_1	Group II intron-encoded protein LtrA
30	ltrA_2	Group II intron-encoded protein LtrA
31	ltrA_3	Group II intron-encoded protein LtrA
32	ltrA_4	Group II intron-encoded protein LtrA
33	ltrA_6	Group II intron-encoded protein LtrA
34	dmlR_8	HTH-type transcriptional regulator DmlR
35	group_2333	HTH-type transcriptional regulator DmlR
36	hyfB_4	Hydrogenase-4 component B
37	lacR_2	Lactose phosphotransferase system repressor
38	tdh_2	L-Threonine 3-dehydrogenase
39	malI_1	Maltose regulon regulatory protein MalI
40	mtlK_2	Mannitol 2-dehydrogenase
41	pglA	N,N′-Diacetylbacillosaminyl-diphospho-undecaprenol alpha-1,3-N-acetylgalactosaminyltransferase
42	group_190	Outer membrane usher protein HtrE precursor
43	group_240	Periplasmic dipeptide transport protein precursor
44	group_2366	Phosphate-starvation-inducible E
45	pduV_2	Propanediol utilization protein PduV
46	nepI_2	Purine ribonucleoside efflux pump NepI
47	group_2	Putative deoxyribonuclease RhsC
48	group_49	Putative fimbrial-like adhesin protein
49	argK_2	Putative GTPase ArgK
50	group_330	Putative HTH-type transcriptional regulator YbbH
51	group_168	Putative lysophospholipase
52	group_2341	Putative oxidoreductase
53	hhoB	Putative serine protease HhoB precursor
54	group_2281	Recombination protein F
55	rbtD	Ribitol 2-dehydrogenase
56	group_2319	Ribokinase
57	araB_1	Ribulokinase
58	hspA_1	Spore protein SP21
59	hspA_2	Spore protein SP21
60	trxC_2	Thioredoxin-2
61	lsrR_1	Transcriptional regulator LsrR
62	group_2327	Type I restriction enzyme EcoKI subunit R
63	hsdR_1	Type I restriction enzyme EcoR124II R protein
64	hsdR_2	Type-1 restriction enzyme R protein
65	group_2288	Tyrosine recombinase XerD
66	group_2310	UDP-glucose 4-epimerase
67	xylB_2	Xylulose kinase
68	group_2360	YfdX protein
69	group_2361	YfdX protein

### Genes involved in biofilm formation

Several genes have been ascribed active roles in biofilm formation in *E. coli* [26–28]. One of the most important is the *flu* gene encoding the antigen 43 protein [29]. In liquid culture, Ag43 leads to autoaggregation and clump formation rapidly followed by bacterial sedimentation. Surprisingly, the *flu* gene was not present in the *E. coli* C genome. We identified a few autotransporter encoding genes, which showed partial homology to Ag43, such as B6N50_05815 (50% similarity over 381aa); however, the homology was too weak to suggest that these genes could play a similar role.

Surface polysaccharides often play an important role in biofilm formation [27, 30]. *E. coli* C forms an O rough R1-type lipopolysaccharide, which serves as a receptor for bacteriophages [3]. Out of the 14 *waa* genes present in *E. coli* K12, we were able to find only 6 in *E. coli* C. Out of the 5 genes *waaA*, *waaC*, *waaQ*, *waaP*, and *waaY*, which are highly conserved and responsible for assembly and phosphorylation of the inner-core region [31], only the first 4 were present in *E. coli* C (Additional file 2: Figure S2). Two remaining genes in *E. coli* C were *waaG*, whose product is an α-glucosyltransferase that adds the first residue (HexI) of the outer core, and *waaF,* which encodes for a HepII transferase [31]. Biofilm formation by a deep rough LPS *hldE* mutant of *E. coli* BW25113 strain was strongly enhanced in comparison with the parental strain and other LPS deficient mutants. The *hldE* strain also showed a phenotype of increased autoaggregation and stronger cell surface hydrophobicity compared to the wild-type [32]. The gene *hldE, which encodes for a* HepI transferase, was found in the *E. coli* C strain. Other mutants in LPS core biosynthesis, which resulted in a deep rough LPS, have been described to decrease adhesion toabiotic surfaces [33]; therefore, we assumed that other genes in this family would not be responsible for the increased biofilm formation by *E. coli* C.

We noticed also that *wzzB,* a regulator of length of O-antigen component of LPS chains was mutated by an IS3 insertion. Another IS insertion was located in UDP-glucose 6-dehydrogenase (B6N50_08940). Both of these genes were located at the end of a long 35 operon-like gene stretch in *E. coli* C, including *wca* operon [34] consisting of 19 genes involved in colanic acid synthesis.

We found that the region involved in biosynthesis of poly-β-1,6-N-acetyl-glucosamine (PGA) was almost 100% identical in both K12 and C strains.

Other types of structures involved in biofilm formation are fimbriae, curli, and conjugative pili [26, 27, 35]. Type 1 pili can adhere to a variety of receptors on eukaryotic cell surfaces. They are well-documented virulence factors in pathogenic *E. coli* and are critical for biofilm formation on abiotic surfaces [36–40]. Type 1 pili are encoded by a contiguous DNA segment, labeled the *fim* operon, which contains 9 genes necessary for their synthesis, assembly, and regulation [41, 42]. In *E. coli* C, almost the entire *fim* operon except the *fimH*, which codes for the mannose-specific adhesin located at the tip of the pilus, was absent and replaced by a type II group integron (Additional file 3: Figure S3). The entire *fim* operon is driven by a single promoter located upstream of the *fimA* gene; therefore, it is possible that the *fimH* gene is not expressed in *E. coli* C. Although we cannot exclude the role of FimH in autoaggregation of *E. coli* C, reports that the function of FimH was inhibited by growth at temperatures at or below 30 °C [43] make it highly unlikely.

Chaperone-usher (CU) fimbriae are adhesive surface organelles typical to many gram-negative bacteria. *E. coli* genomes contain a large array of characterized and putative CU fimbrial operons [44]. Korea at al. characterized the *ycb*, *ybg*, *yfc*, *yad*, *yra*, *sfm,* and *yeh* operons of *E. coli* K-12, which display sequence and organizational similarities to type 1 fimbriae exported by the CU pathway [45]. They showed that, although these CU operons were not well expressed under laboratory conditions, 6 of them were nevertheless functional when expressed and promote attachment to abiotic and/or epithelial cell surfaces [45]. A total of 10 CU operons have been identified in *E. coli* K12 MG1655 [44]. We identified all 10 CU operons in the *E. coli* C genome. Furthermore, we found that the IS5 insertion in the K12 *yhcE* gene was not present in *E. coli* C (Additional file 4: Figure S4A). We also noticed that two insertion sequences were inserted in the *yad* region (Additional file 4: Figure S4B).

Curli are another proteinaceous extracellular fiber involved in surface and cell-cell contacts that promote community behavior and host cell colonization [46]. Curli synthesis and transport are controlled by two operons, *csgBAC* and *csgDEFG*. The *csgBA* operon encodes the major structural subunit CsgA and the nucleator protein CsgB [47]. CsgC plays a role in the extracellular assembly of CsgA. In the absence of CsgB, curli are not assembled and the CsgA - main subunit protein, remains unpolymerized when secreted from the cell [46]. The *csgDEFG* operon encodes 4 accessory proteins involved in assembly of curli. The *csgBA* operon is positively regulated by transcriptional regulator CsgD [47]. We found that the intergenic region between *csgBA* and *csgDEFG* has been modified in *E. coli* C. An IS5/IS1182 family transposase was inserted between 106 bp upstream of the *csgD* gene and 96 bp inside the *csgA* gene (Additional file 5: Figure S5). The entire *csgB* gene as well as the first 32aa of CsgA have been deleted. The full CsgA protein in *E. coli* K12 contains 151aa while the truncated version in strain C consisted of only 107aa and might not be expressed. Furthermore, *csgD* expression is driven by a promoter located ~ 130 bp upstream [48, 49]. The IS5/IS1182 family transposase inserted between that promoter and the *csgD* gene was transcribed in the same direction, so it might not cause a polar mutation but definitely would interfere with the sophisticated regulation of *csgD* expression by multiple transcription factors [48, 49]. As *E. coli* C did not carry any extrachromosomal DNA, conjugative pili, which usually play an important role in biofilm formation [50], were not analyzed.

Biofilm formation is a bacterial response to stressful environmental conditions [9]. This response requires an orchestra of sensors and regulators during each step of the biofilm formation process. We analyzed a few of the most important mechanisms, such as CpxAR, RcsCD, and EnvZ/OmpR [27]. In all three cases, we observed the same gene structure and a high degree of DNA sequence identity between the *E. coli* C and K12 strains.

**Fig. 8:**
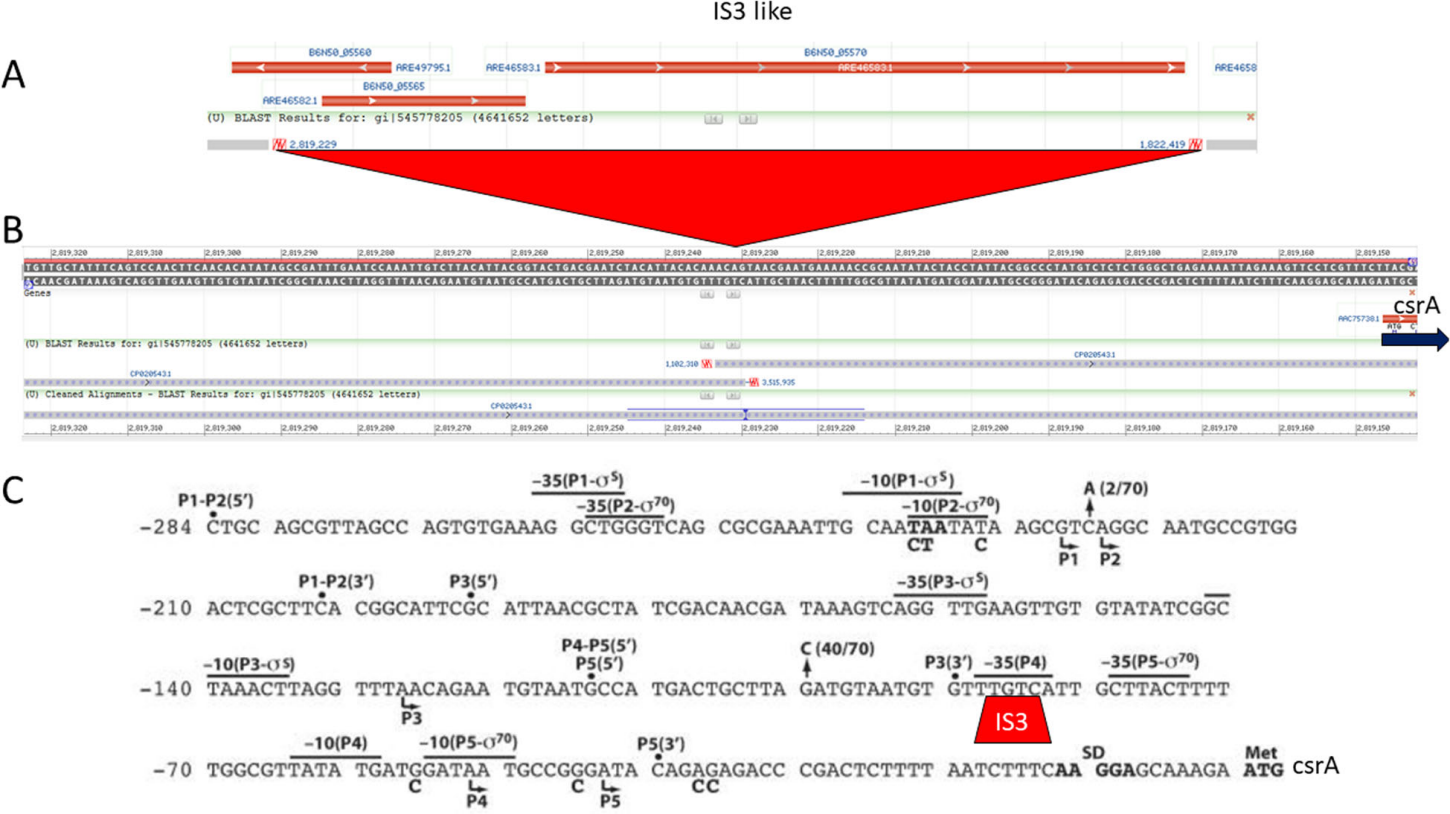
Insertion of IS3-like sequence in the promoter region of *csrA* gene. **a** Structure of the IS3 like sequence; (**b**) genome **v**iew of K12 (upper) *csrA* promoter region BLAST results with *E. coli* C; (**c**) *csrA* promoter region [53] with the IS3 insertion site

Another regulatory loop includes the carbon storage regulator *csrA* and its small RNAs [51]. Mutations within the *csrA* gene induced biofilm formation in many bacteria [17, 51]. Recently, the CsrA regulation has been connected with multiple other transcription factors, including NhaR, UvrY, SdiA, RecA, LexA, Hns, and many more [20, 52]. The regulatory loop with NhaR protein drew our attention as it is responsible for integrating the stress associated with high salt/high pH and low temperature [20]. We found that the *nhaAR* and *sdiA*/*uvrY* regions of *E. coli* C were almost identical with the corresponding regions in the K12 strain. We amplified and sequenced the *csrA* gene from the *E. coli* C strain to verify its presence and integrity (Additional file 6: Figure S6). Detailed analysis of the *csrA* region revealed the presence of an IS3-like insertion sequence 86 bp upstream of the ATG codon (Fig. 8). The *csrA* gene is driven by 5 different promoters [53]. The distal (− 227 bp) promoter P1 is recognized by sigma^70^ and sigma^32^ factors and enhanced by DskA. The P2 (− 224 bp) promoter depends on sigma^70^. Both the P1 and P2 promoters are relatively weak promoters [53]. The P3 promoter is located 127 bp upstream of *csrA* and it is recognized by the stationary RpoS (sigma^32^) polymerase. This promoter is the strongest promoter of *csrA* gene. Promoters P4 and P5 are located 52 bp and 43 bp, respectively, upstream of the *csrA* gene. These promoters are driven by the sigma^70^ polymerase and are active mainly during exponential growth [53]. The IS3 insertion was located within the − 35 region of the P4 promoter. That location should almost completely abolish expression of the *csrA* gene in the stationary phase of bacterial growth and probably was the main reason for increased biofilm production by the *E. coli* C strain. Both small RNAs, *csrB* and *csrC,* which regulate CsrA activity, were found unchanged in the *E. coli* C genome.

### Confirmation of IS3 insertion and its complementation by overexpression of *csrA* gene

First we compared the biofilm formation ability of *E. coli* C and the K12 *csrA* mutant. The 72-h-old biofilms of both strains formed on microscope slides were similar (Additional file 7: Figure S7A). The 24-h 96-well plate biofilm assay showed that at 37 °C the K12 *csrA* mutant formed 30% more biofilm than *E. coli* C (*p* = 0.001, Student t-test). At 30 °C strain C produced more biofilm, but the difference was not statistically significant (Additional file 7: Figure S7B), although the *csrA* mutant aggregated ~ 56% more efficiently than the *E. coli* C strain in the same conditions. To confirm the presence of the IS3 insertion in the *csrA* promoter region, we designed PCR primers specific for the *alaS*-*csrA* intergenic region. Amplification results confirmed the presence of IS3 in the *E. coli* C promoter region (Additional file 8: Figure S8).

**Fig. 9:**
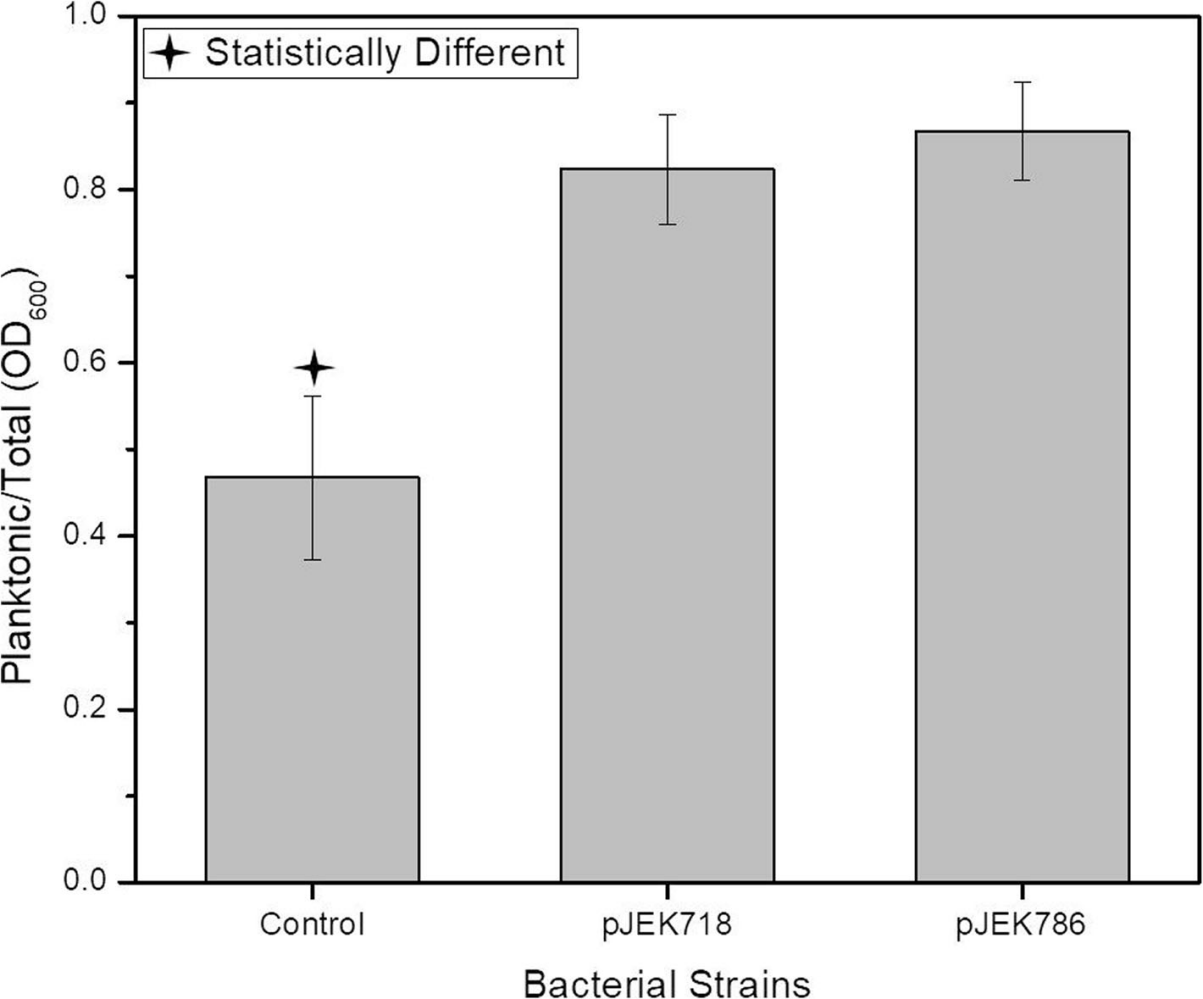
Complementation of *E. coli* C aggregation phenotype by introduction of pJEK718 and pJEK786 plasmids overexpressing the CsrA protein

To see if extrachromosomal expression of the CsrA protein affects the aggregation phenotype, we cloned the *csrA* gene downstream of a *plac* promoter in pBBR1MCS-5 [54], resulting in plasmid pJEK718 or downstream of the constitutive *pcat* (chloramphenicol) promoter in pJEK786. Plasmids were transformed into *E. coli* C strain and the resulting clones were grown in LB Miller broth (30 °C, 250 rpm). The results showed that the ratio of planktonic to total cells in *E. coli* C carrying both constructs overexpressing the *csrA* gene was ~ 1.8 times higher (*f*-ratio = 78.12363, *p* < 0.00001) than in the control carrying the non-recombined vector (Fig. 9). We also noticed that the control strain showed a slightly higher amount of planktonic cells than the plasmidless control (shown on Fig. 3) (0.46 vs. 0.36), although the difference was not statistically significant (*p*  = 0.09, Student t-test).

### Expression of *csrA* promoter in *E. coli* K12 and *E. coli* C

To analyze activities of the *csrA* promoter from *E. coli* C, we cloned PCR products containing sequences upstream of the *csrA* gene (Additional file 8: Figure S8) into a pAG136 plasmid vector carrying promoterless EGFP-YFAST reporters (pJEKd1750) [55]. The *E. coli* C *csrA* promoter was overexpressed in both strains however, the promoter activity was much stronger in the native strain than in K12 (Additional file 9: Figure S9). We notice that the highest differences (3.2 and 2.4, at 37 °C and 30 °C, respectively) occurred at the late exponential phase (~ 4.5 h and ~ 10 h) (Additional file 9: Figure S9). We noticed that the presence of an additional copy of *pcsrA* in a high copy number plasmid induced aggregation of *E. coli* C at 37 °C. The ratio of planktonic/total cells was similar (Additional file 10: Figure S10) to that obtained for the parental *E. coli* C strain at 30 °C (Figs. 2 and 3) (0.36 and 0.35, respectively).

**Fig. 10:**
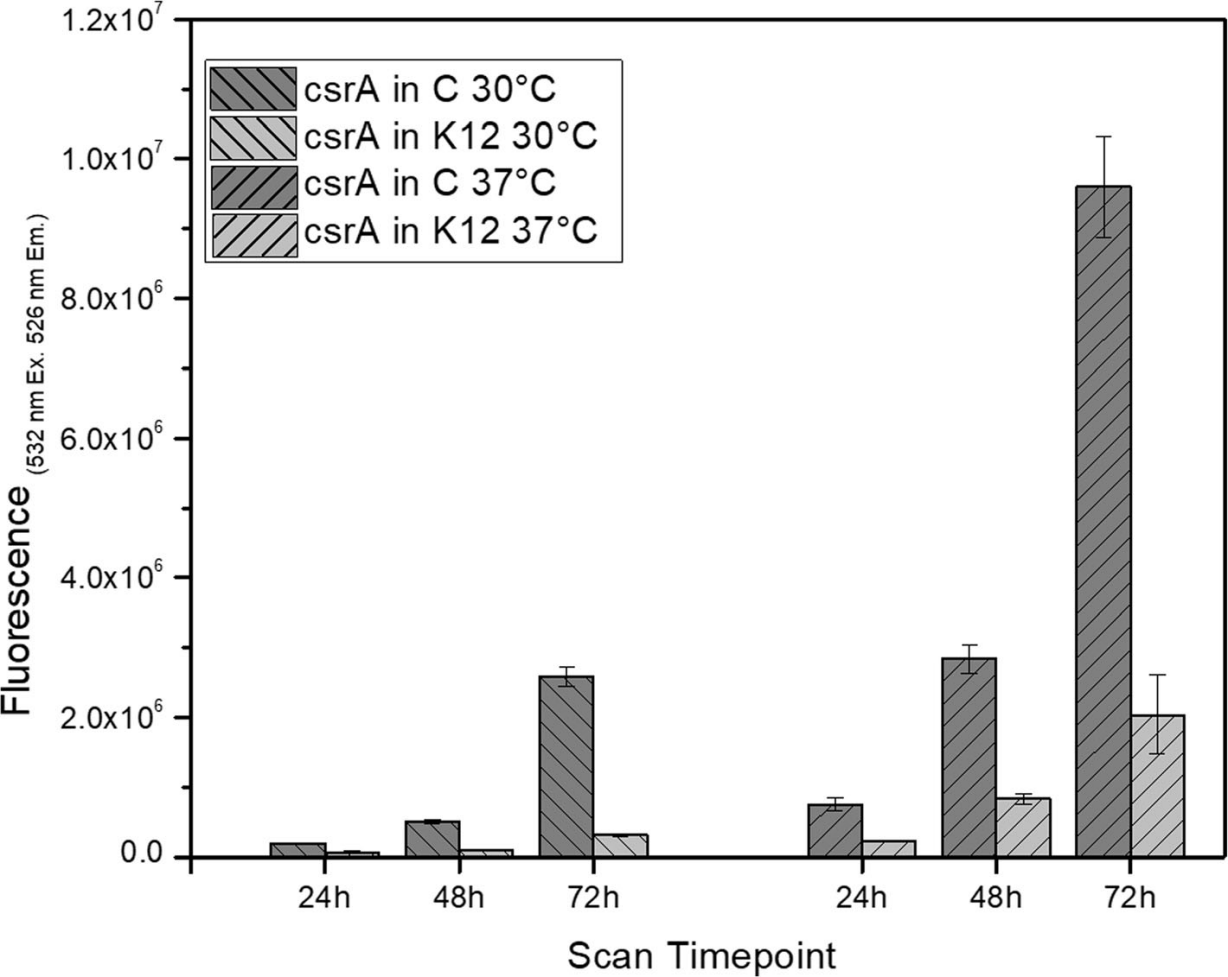
Activity of *pcsrA* promoter (pJEKd1571) in 24 h, 48 h and, 72 h old colonies of *E. coli* C and K12 grown at 30 °C and 37 °C on LB Miller agar plates. Data represents the mean values from 3 biological replicates each containing 3 colonies. Differences between strains at all time points and conditions were statistically significant

**Fig. 11:**
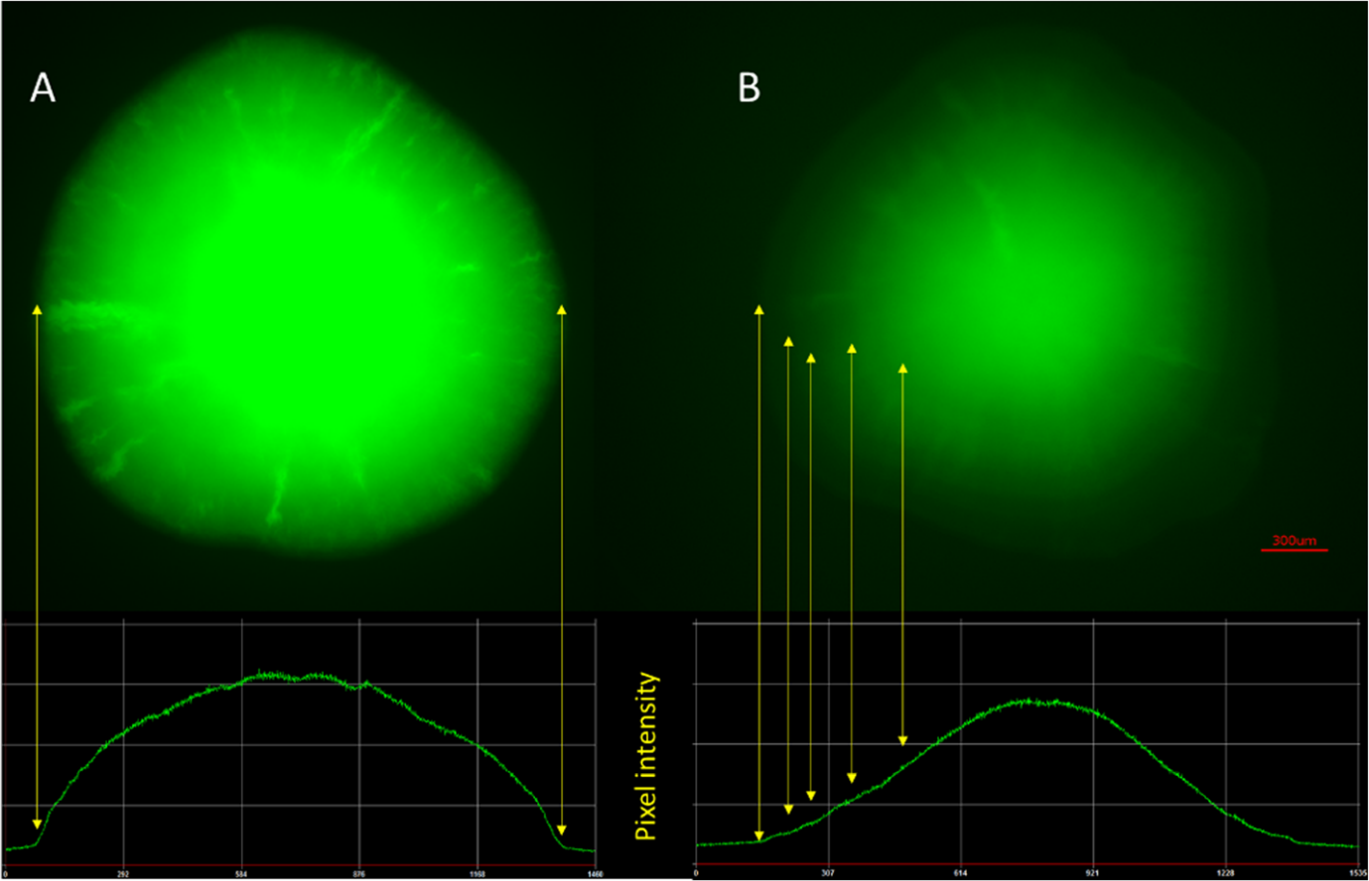
Microscopic picture of the 72 h old *E. coli* C (**a**) and K12 (**b**) colonies containing pJEKd1571 grown at 37 °C on LB Miller agar plates. Pixel intensity plots for each colony are shown below. Yellow arrows show the colony borders and distinct *pcsrA* expression intensities

The aggregation phenotype was correlated with the highest *pcsrA* activity at the entrance to the stationary phase (data not shown). As the aggregation might affect the measurements we decided to use a colony assay to measure the promoter activity over the long time. The LB agar plates with spots of *E. coli* C and K12 carrying pJEKd1751 reporter plasmids with a short half-life form of GFP [ASV] were incubated at 30 °C and 37 °C and the fluorescence activity was measured by a Typhoon 9400 Variable Mode Imager (Fig. 10). The data showed an increased *pcsrA* activity over the 72 h time period in both strains with much higher activity in the native *E. coli* C strain (Fig. 10). The highest differences between the two strains, 8.15 and 4.71, were observed at 72 h at 30 °C and 37 °C, respectively (Fig. 10). As the half-life of the GFP [ASV] is only 110 min [56], we concluded that in the K12 strain *pcsrA* promoter was active mostly at the stationary phase while in the *E. coli* C its activity was quasi constitutive, but also enhanced at the stationary phase (Fig. 10). To test that hypothesis we analyzed the spatial expression of the *pcsrA* promoter in 72 h old bacterial colonies using a fluorescence microscope (Fig. 11). The pictures fully supported our premises. In the *E. coli* C the entire colony showed an intensive fluorescence with the highest level in the center (Fig. 11a). In the K12 strain we noticed 5 discrete zones with different fluorescence activities (Fig. 11b). The edge of the colony, which should consist of the youngest, still dividing and metabolically active cells, showed the lowest, while the center of the colony with the oldest cells showed the highest fluorescence (Fig. 11b).

### Location of IS3-like insertions in *E. coli* C genome and role of ISs in biofilm gene expression

Based on the *E. coli* C *pcsrA* promoter structure in comparison to the *pcsrA*-K12 [53] and its transcriptional activities, we concluded that the small 80-bp region containing the P4 and P5 promoters could not be solely responsible for the *csrA* transcription. Insertion sequences play a huge role in bacterial genome evolution [57]. They can also insert upstream of a gene and activate its expression [58]. Out of 177 genes that were unique to *E. coli C,* 55 encoded transposases (Additional file 11: Table S1).

Using BLAST, we found that the IS3-like sequence present in front of the *csrA* gene was present in 19 other locations throughout the genome (data not shown). Analyzing these locations, we found that in 12 cases the IS3 might drive the expression of downstream located genes (Table. 2). One of the most striking observations was that the IS3-like sequence was located in front of an alternative sigma^70^ factor, which was not present in the K12 strain (Table. 2). Based on the *pcsrA* expression, we concluded that a promoter located inside the IS3 drives permanent expression of the following genes. The presence of the constitutively expressed alternative sigma^70^ factor in the *E. coli* C can drive expression of the sigma^70^ promoters in a growth phase independent manner. As the remaining *E. coli* C *csrA* promoters P4 and P5 are sigma^70^ dependent promoters [53], it might explain their strong activity along all the cell growth phases. Further studies will be conducted to prove that hypothesis.

**Table 2:** Genes located downstream of the IS3-like element

	Gene name	Function
1.	B6N50_06210–15 (2 genes)	Unknown function and small toxic protein ShoB
2	B6N50_07905–890 (4 genes)	Acetate CoA-transferase subunit alpha, acetate CoA-transferase subunit beta, short-chain fatty acids transporter and acetyl-CoA acetyltransferase
3	B6N50_12005–12,000 (2 genes)	Restriction endonuclease (pseudogene- missing start), RNA polymerase subunit sigma-70 (pseudogene- missing stop)
4	B6N50_11960–55 (2 genes)	Hypothetical protein, hypothetical protein
5	B6N50_05830 (1 gene)	Hypothetical protein
6	B6N50_05870 (1 gene)	DEAD/DEAH box helicase
7	B6N50_10145 (1 gene)	Hypothetical protein
8	B6N50_11585 (1 gene)	Hypothetical protein
9	B6N50_11780–85 (2 genes)	TIGR00156 family protein, sensor domain-containing diguanylate cyclase
10	B6N50_11960 (1 gene)	Hypothetical protein
11	B6N50_12235–260 (6 genes)	NarK family nitrate/nitrite MFS transporter, nitrate reductase subunit alpha, nitrate reductase subunit beta, nitrate reductase molybdenum cofactor assembly chaperone NarW, respiratory nitrate reductase subunit gamma, hypothetical protein
12	B6N50_19300–320 (5 genes)	Outer membrane usher protein, fimbrial protein, fimbrial protein StaE, fimbrial protein StaF, hypothetical protein

## Discussion

*E. coli* is the most common bacterial research model organism. Out of the five strains used only *the E. coli* C genome has not been sequenced. Here, we sequenced and analyzed the *E. coli C* genome and revealed its specific features that lead to enhanced biofilm formation. Recently, a new *E. coli* strain C genome has been submitted to the GenBank database (CP029371.1). Homology search revealed that this strain was not closely related to our strain. However, the sequence homology search of GenBank available *E. coli* genomes revealed that two isolates, WG5 (CP024090.1) and NTCT122 (LT906474.1), showed identical *csrA* promoter regions. Strain WG5 is in fact an *E. coli* C derivative resistant to nalidixic acid [59, 60]. This *E. coli* C, also known as strain CN, is publicly available in the ATCC (ATCC number 700078). We found that our sequence is very similar to the WG5 sequence, although the inverted 300 kb region between 107 and 407 kb was not present in WG5. Also some of the insertion sequences were not present in the WG5 genome. These findings again revealed a role of different mobile elements in genome rearrangements and evolution. As the bacterial genome undergoes a constant evolution and adaptation [61] and bacterial mobile elements are the most common mechanism of those processes [62, 63], one may ask why in this particular strain, unlike the other laboratory strains, the selection toward planktonic cells did not take place. There is no simple answer; however, we can speculate that as this strain is used for proliferation of bacteriophages the fact that phages kill planktonic cells might reduce the selection toward free floating cells. The second hypothesis is that for bacteriophage research using the *E. coli* C, the ATCC recommends low-salt (0.5% NaCl) or no salt Nutrient (#139) broth medium. As we showed, the low-salt medium reduced bacterial stress and most likely reduced the level of genome rearrangements, keeping the natural properties for biofilm formation characteristic for the wild-type strains in this laboratory *E. coli* C strain.

## Conclusions

Biofilms are the most prevalent form of bacterial life [9, 30] and as such have drawn significant attention from the scientific community over the past quarter century. However, only in 2018 did the number of biofilm related articles reach 24,000, based on a Google Scholar search. As in all other fields, biofilm research needs to develop and follow standard protocols and methods that can be used in different laboratories and give comparable results. Unfortunately, a standardized methodological approach to biofilm models has not been adopted, leading to a large disparity among testing conditions. This has made it almost impossible to compare data across multiple laboratories, leaving large gaps in the evidence [64]. In our work, we described and characterized biofilm formation in the classic laboratory strain, *E. coli* C [2, 65]. We have used that strain in our biofilm-related research for almost a decade and we would like to share it with the biofilm community and propose to use it as a model organism in *E. coli*-based biofilm-related research.

## Methods

### Bacterial strains and growth conditions

Bacterial strains are listed in Table 3. Strains were grown in M9 with glycerol medium or LB Miller, LB Lennox, or modified Lennox with 0.75% NaCl broth with appropriate antibiotics, kanamycin (Km-50 μg/ml), gentamycin (Gm-10 μg/ml), and chloramphenicol (Cm-30 μg/ml).

**Table 3:** Bacterial strains used in this work

Strain	Genotype	Source
*E. coli* C	WT	Holly A. Wichman, James Bull, UofI, UT.
*E. coli* K12 MG1655	WT	Lab collection
*E. coli* Crooks	WT	Lonnie Ingram, UF
*E. coli* B	WT	ATCC
*E. coli* W	WT	ATCC
*E.coli* K12 csrA	csrA::mini-Tn5 Km^R^	Tony Romeo, UF
*E. coli* K12 MG1655 EC100	F^−^*mcr*A ∆(*mrr-hsdRMS-mcrBC*) φ80d*lac*Z∆M15 ∆*lac* X74 *recA*1 *endA*1 *araD*139 ∆(*ara, leu*)7697 *galU galK* λ–*rpsL nupG*.	Lucigen
Plasmids		
pBBR1MCS-5	Broad host range mobilizable plasmid, Gm^R^	[54]
pAG136	pET28B –EGFP-YFAST, Km^R^ promoter probe vector	[55]
pProbe GFP [ASV]	Short-life GFP promoter probe vector, Km^R^	[56]
pJEKd1750	1638-bp fragment with *E.coli* C IS3-*csrA* promoter in BglII/XbaI sites of pAG136, Km^R^	This work
pJEKd1751	1638-bp fragment with *E. coli* C IS3-*csrA* promoter in SmaI site of pProbe GFP [ASV], Km^R^	This work
pJEK718	*csrA* gene driven by the *plac* promoter in pBBR1MCS-5, Gm^R^	This work
pJEK786	*csrA* gene driven by the *pcat* promoter in pBBR1MCS-5, Gm^R^ Cm^R^	This work

Abbreviations: Km^R^, Gm^R^_,_ Cm^R^ – kanamycin, gentamycin, and chloramphenicol resistance

### Biofilm assays

Biofilms on microscope slide were grown as described previously [19]. For biofilm formation on a polystyrene surface, flat-bottom 96-well microtiter plates (Corning Inc.) were used [18]. *E. coli* overnight cultures were diluted 1:40 in fresh medium, and 150-μL aliquots were dispensed into wells. After 24 h of incubation (37 °C), cell density was measured (OD_600_) using a plate reader, and 30 μL of Gram Crystal Violet (Remel) was applied for staining for 1 h. Plates were washed with water and air dried, and crystal violet was solubilized with an ethanol-acetone (4:1) solution. The OD_570_ was determined from this solution, and the biofilm amount was calculated as the ratio of OD_570_ to OD_600_ [19].

### Construction of CsrA overexpressing strain

A 277-bp DNA fragment containing the *csrA* gene was amplified using csrAF-aaa GAATTCGTAATACGACTCACTATAGGGTTTC csrAR –aaaGAATTCTTTGAGGGTGCGTCTCACCGATAAAG primers. This fragment was cloned directly into the *Eco*RI site of the pBBR1MCS-5 vector [54]. Sequence orientation was verified by DNA sequencing and the correct clone with *csrA* gene downstream of the *plac* promoter was named pJEK718. To express the *csrA* gene with a constitutive *pcat* (chloramphenicol) promoter, a PCR amplified *cat* gene (870 bp, catF-aaaGATCCTGGTGTCCCTGTTGATACCGGGAA; cat-R-aaa GGATCCCCCAGGCGTTTAAGGGCACCAATAAC) was cloned in the *Bam*HI site of one of the clones that carried the *csrA* gene in the orientation opposite to the *plac* promoter in the pBBR1MCS-5 vector. Selection for Cm-resistant clones ensured the promoter activity and the correct orientation was verified by PCR with catF/csrAR primers and DNA sequencing. The correct plasmid was named pJEK786. Plasmids were introduced into the *E. coli* C strain by TSS transformation [66].

### Confirmation of IS3 insertion and construction of GFP reporter fusions

PCR fragments containing the *csrA* promoter were amplified using pcsrA aaaagatctCTGATTGCAGGCGTATCTAAGG and pcsrAR aaatctagaAAAGATTAAAAGAGTCGGGTCTCTCTGTATCC primer pair from both *E. coli* K12 and C strains and cloned into the BglII/XbaI site of the pAG136 plasmid [55] or the *Sma*I site of the pPROBE-GFP [LVA] promoter probe vector [56]. All constructs were verified by DNA sequencing. Plasmids were introduced into both the *E. coli* K12 and C strains by a TSS transformation [66]. GFP activity (OD_480–520_) was measured using BioTek Synergy HT (BioTek) or Tecan InfiniteM200 Pro (Tecan) plate readers and normalized to the optical density of the culture (OD_600_), yielding relative fluorescence units (RFU; FL_480–520_/OD_600_). For quantification of promoter activities in late stationary phase, single colonies were inoculated into 5 mL of LB broth, vortexed and 5 μL of cell suspension was spotted on LB Miller agar plates. Plates were incubated at 30 °C or 37 °C. At the specific time points, plates were scanned with a Typhoon 9400 Variable Mode Imager using 532/526-nm excitation/emission wavelengths (GE Healthcare). Scans were analyzed using the ImageQuant TL software (GE Healthcare). Student t-test was used to compare results and check statistical significance. Fluorescence microscopy was done with a Keyence BZ-X710 All-in-One Fluorescence microscope (Keyence).

### Cell aggregation experiments

*E. coli* strains were grown in LB Miller broth at 30 °C in shaking conditions (250 rpm). One milliliter of the culture was transferred to standard polypropylene spectrophotometer cuvettes to measure planktonic cells densities (OD_600_). Remaining cultures were vortexed ~ 1 min and 1 mL was aliquoted into cuvettes to measure the total cell densities (OD_600_). Aggregation was calculated as a ratio of planktonic to total cell density. For the aggregation experiment, overnight cultures were vortexed ~ 1 min and 1 mL was aliquoted into standard polypropylene spectrophotometer cuvettes and capped. Cuvettes were incubated statically at 12 °C, 24 °C (room temperature), and 37 °C. Cell densities were measured every hour by measuring OD_600_.

### DNA sequencing and sequence analyses

DNA for sequencing was isolated using the Qiagen Blood and Tissue DNA Isolation Kit. Genomic DNA was mechanically sheared using a Covaris g-TUBE. The SMRTbell template preparation kit 1.0 (Pacific Biosciences, Menlo Park, CA, USA) was used according to the PacBio standard protocol (10-kb template preparation using the BluePippin size-selection system Sage Science). After SMRTbell preparation and polymerase binding, the libraries were loaded on SMRTcells via magbead loading and run on a PacBio RS II instrument (Pacific Biosciences) using a C4 chemistry. DNA sequence data were assembled by the HGAP Assembly 2 and annotated by Prokka or NCBI’s Prokaryotic Genome Automatic Annotation Pipeline (PGAAP) [67]. *E. coli* C, K12, B, W, and Crook genomes were analyzed by Roary, Mauve, and Geneious R11.

### Optical mapping - high molecular weight DNA extraction

Cells from overnight culture were washed with PBS, resuspended in cell resuspension buffer, and embedded into low-melting-point agarose gel plugs (BioRad #170–3592, Hercules, CA, USA). Plugs were incubated with lysis buffer and proteinase K for 4 h at 50 °C. Plugs were washed, melted, and solubilized with GELase (Epicentre, Madison, WI, USA). Purified DNA was subjected to 4 h of drop-dialysis and DNA concentration was determined using Quant-iTdsDNA Assay Kit (Invitrogen/Molecular Probes, Carlsbad, CA, USA). DNA quality was assessed with pulsed-field gel electrophoresis. High molecular weight DNA was labeled according to commercial protocols with the IrysPrep Reagent Kit (Bionano Genomics). Roughly 300 ng of purified genomic DNA was nicked with 7 U of nicking endonuclease Nt.BspQI (New England Biolabs -NEB) at 37 °C for 2 h in NEB Buffer 3. Nicked DNA was labeled with a fluorescent-dUTP nucleotide analog using Taq polymerase (NEB) for 1 h at 72 °C. Nicks were repaired with Taq ligase (NEB) in the presence of dNTPs. The backbone of fluorescently labeled DNA was stained with YOYO-1 (Invitrogen). Labeled DNA molecules entered nanochannel arrays of an IrysChip (Bionano Genomics) via automated electrophoresis. Molecules were linearized in the nanochannel arrays and imaged. An in-house image detection software detected the stained DNA backbone and locations of fluorescent labels across each molecule. The set of label locations within each molecule defined the single-molecule maps. The *E. coli strain C* reference sequence was in silico nicked with Nt.BspQI. Raw single-molecule maps were filtered by minimum length of 150 kbp. Molecule maps were aligned to the *E. coli* reference map with OMBlast. OMBlast is an optical mapping alignment tool using a seed-and-extend approach and allows split-mapping [68]. Alignments were performed with the OMBlastMapper module (version 1.4a) using the following parameters: --writeunmap false --optresoutformat 2 --falselimit 8 --maxalignitem 2 --minconf 0. Molecule maps with partial alignments to regions flanking the putative insertion breakpoint coordinates were extracted from the alignment output file. Molecule maps were manually inspected for label matches in segments 5′ and 3′ to the putative inverted region and into the inversion. The non-aligned segments of these maps, which extended into the inverted region with label matches to the opposing side in a reverse fashion, were retained.

### Statistical analysis

Statistical analysis was carried out in the R computing environment and in Graphpad. One-way ANOVA was calculated using an online tool (https://www.socscistatistics.com/tests/anova/default2.aspx) or R package. Relevant statistical information is included in the methods for each experiment. Error bars show standard deviation from the mean. Asterisks represent statistical significance at *p* < 0.05.

## Supplementary information

**Additional file 1: Figure S1.** Maximal likelihood tree based on gene homology within five *E. coli* strains.

**Additional file 2: Figure S2.** Genome view of (A) K12 LPS (*waa*) regions BLAST results with *E. coli* C genome and (B) colonic acid (*wca*) region in *E. coli* C. Red double-headed arrow shows deleted region in strain C. Green arrow indicates a long operon like stretch of 35 genes with IS3 insertions in *wzzB* and B6N50_08940 genes (black double-headed arrows).

**Additional file 3: Figure S3.** Genome view of K12 *fim* region BLAST results with *E. coli* C genome. Red double-headed arrow shows deleted region in strain C.

**Additional file 4: Figure S4.** Genome view of K12 *yhc* region BLAST results with *E. coli* C genome (A) and the *E. coli* C *yad* region with two IS insertions (black arrows). Deletion of IS5 in *E. coli* C *yhcE* gene is highlighted.

**Additional file 5: Figure S5.** Genome view of K12 (upper) and C strain (lower) *csg* region BLAST results with *E. coli* C genome. Red double-headed arrow shows region replaced by IS5 in strain C.

**Additional file 6: Figure S6.** PCR amplification of the *csrA* gene from *E. coli* C and K12 strains.

**Additional file 7: Figure S7.** Biofilm formation by *E. coli* C and K12 *csrA* mutant strains on (A) microscope slides (LB medium- 72 h) and (B) 96-well plates (LB Miller broth 37 °C; 24 h).

**Additional file 8: Figure S8.** PCR amplification of the *alaS-csrA* intergenic region from *E. coli* C and K12 strains.

**Additional file 9: Figure S9.** Differences in relative *pcsrA* promoter activity between *E. coli* C and K12 strains grown in LB Miller broth at 24 °C and 37 °C (250 rpm). Cell densities (OD_600_) and fluorescence (480 nm Ex./520 nm Em.) were measured over the time course to show the relative promoter activity in each strain and condition. The graph represents the ratios between these activities in *E. coli* C and K12 strains at the specific time points.

**Additional file 10: Figure S10.** Cell aggregation of *E. coli* C and K12 carrying the pJEKd1750 plasmid in overnight culture grown at 37 °C in LB Miller broth on shaker at 250 rpm. Ratio of planktonic cells to total cells measured as OD_600_.

**Additional file 11: Table S1.** Gene content comparison between five *E. coli* strains.

## Data Availability

All other relevant data are available in this article and its Supplementary Information files. The complete genome of *E. coli* C has been deposited in GenBank under accession no. CP020543.1.

## Abbreviations

ATCC: American Type Culture Collection
BLAST: Basic Local Alignment Search Tool
Bp/kbp: Base Pair/Kilo Base Pair
CU: Chaperone-Usher
DNA: DeoxyriboNucleic Acid
dNTPs: Deoxy Nucleotides
EGFP-YFAST: Enhanced GFP- Yellow Fluorescence-Activating and absorption-Shifting Tag
GFP [ASV]: short half-life form of GFP
GFP: Green Fluorescence Protein
h: Hours
IS: Insertion Sequence
LB: Luria-Bertani (Lysogenic Broth) medium
OD: Optical Density
p: Promoter
PCR: Polymerase Chain Reaction
RFU: Relative Fluorescence Units
RNA: RiboNucleic Acid
rpm: Rotations Per Minute
TSS: Transformation and Storage Solution
UDP: Uridine DiPhosphate

## References

[1] Lieb M, Weigle JJ, Kellenberger E (1955). A study of hybrids between two strains of Escherichia coli. J Bacteriol.

[2] Bertani G, Weigle JJ (1953). Host controlled variation in bacterial viruses. J Bacteriol.

[3] Feige U, Stirm S (1976). On the structure of the Escherichia coli C cell wall lipopolysaccharide core and on its phiX174 receptor region. Biochem Biophys Res Commun.

[4] Wiman M, Bertani G, Kelly B, Sasaki I (1970). Genetic map of Escherichia coli strain C. Mol Gen Genet.

[5] Link C, Reiner A (1983). Genotypic exclusion: a novel relationship between the ribitol-arabitol and galactitol genes of E. coli. Mol Gen Genet.

[6] Carzaniga T, Antoniani D, Dehò G, Briani F, Landini P (2012). The RNA processing enzyme polynucleotide phosphorylase negatively controls biofilm formation by repressing poly-N-acetylglucosamine (PNAG) production in Escherichia coli C. BMC Microbiol.

[7] Davey ME, Toole GA (2000). Microbial Biofilms: from Ecology to Molecular Genetics. Microbiol Mol Biol Rev.

[8] Tolker-Nielsen T. Biofilm Development Microbiology Spectrum. 2015:3(2). 10.1128/microbiolspec.MB-0001-2014 26104692

[9] Costerton JW, Lewandowski Z, Caldwell DE, Korber DR, Lappin-Scott HM (1995). MICROBIAL BIOFILMS. Annu Rev Microbiol.

[10] Ehrlich GD, Stoodley P, Kathju S, Zhao Y, McLeod BR, Balaban N (2005). Engineering approaches for the detection and control of orthopaedic biofilm infections. Clin Orthop Relat Res.

[11] Visick KL, Schembri MA, Yildiz F, Ghigo J-M (2016). Biofilms 2015: multidisciplinary approaches shed light into Microbial life on surfaces. J Bacteriol.

[12] Rossi E, Cimdins A, Lüthje P, Brauner A, Sjöling Å, Landini P (2018). “It’s a gut feeling” – Escherichia coli biofilm formation in the gastrointestinal tract environment. Crit Rev Microbiol.

[13] Michalik M, Samet A, Marszałek A, Krawczyk B, Kotłowski R, Nowicki A (2018). Intra-operative biopsy in chronic sinusitis detects pathogenic Escherichia coli that carry fimG/H, fyuA and agn43 genes coding biofilm formation. PLoS One.

[14] Patel JK, Perez Oa Fau-Viera MH, Viera Mh Fau-Halem M, Halem M Fau-Berman B, Berman B. Ecthyma gangrenosum caused by *Escherichia coli* bacteremia: a case report and review of the literature. Cutis. 2009;84(5)(0011–4162 (Print)):261–267. 20099619

[15] Kim S-H, Kwon J-C, Choi S-M, Lee D-G, Park SH, Choi J-H (2013). Escherichia coli and Klebsiella pneumoniae bacteremia in patients with neutropenic fever: factors associated with extended-spectrum β-lactamase production and its impact on outcome. Ann Hematol.

[16] Yoshikawa Akikazu, Isono Setsuko, Sheback Abraham, Isono Katsumi (1987). Cloning and nucleotide sequencing of the genes rimI and rimJ which encode enzymes acetylating ribosomal proteins S18 and S5 of Escherichia coli K12. MGG Molecular & General Genetics.

[17] Romeo T, Gong M, Liu MY, Brun-Zinkernagel AM (1993). Identification and molecular characterization of csrA, a pleiotropic gene from Escherichia coli that affects glycogen biosynthesis, gluconeogenesis, cell size, and surface properties. J Bacteriol.

[18] O'Toole GA (2011). Microtiter dish biofilm formation assay. JoVE..

[19] Król JE, Wojtowicz AJ, Rogers LM, Heuer H, Smalla K, Krone SM (2013). Invasion of E. coli biofilms by antibiotic resistance plasmids. Plasmid..

[20] Król JE (2018). Regulatory loop between the CsrA system and NhaR, a high salt/high pH regulator. PLoS One.

[21] Blattner FR, Plunkett G, Bloch CA, Perna NT, Burland V, Riley M (1997). The Complete Genome Sequence of &lt;em&gt;*Escherichia coli*&lt;/em&gt; K-12. Science..

[22] Archer CT, Kim JF, Jeong H, Park JH, Vickers CE, Lee SY (2011). The genome sequence of *E. coli* W (ATCC 9637): comparative genome analysis and an improved genome-scale reconstruction of *E. coli*. BMC Genomics.

[23] Jeong H, Barbe V, Lee CH, Vallenet D, Yu DS, Choi S-H (2009). Genome sequences of Escherichia coli B strains REL606 and BL21(DE3). J Mol Biol.

[24] Lam ET, Hastie A, Lin C, Ehrlich D, Das SK, Austin MD (2012). Genome mapping on nanochannel arrays for structural variation analysis and sequence assembly. Nat Biotechnol.

[25] Alexander Lex, Nils Gehlenborg, Hendrik Strobelt, Romain Vuillemot, Pfister H. UpSet: Visualization of Intersecting Sets. IEEE Transactions on Visualization and Computer Graphics (InfoVis '14). 2014;20(12):1983--92. 10.1109/TVCG.2014.2346248 PMC4720993 26356912

[26] Pratt LA, Kolter R. Genetic analyses of bacterial biofilm formation. Mol Microbiol. 1998 30(2)(1369–5274 (Print)):285–93. 10.1046/j.1365-2958.1998.01061.x 9791174

[27] Beloin C, Roux A, Ghigo JM (2008). Escherichia coli biofilms. Curr Top Microbiol Immunol.

[28] Niba ETE, Naka Y, Nagase M, Mori H, Kitakawa M (2007). A genome-wide approach to identify the genes involved in biofilm formation in E. coli. DNA res.

[29] Diderichsen B (1980). flu, a metastable gene controlling surface properties of *Escherichia coli*. J Bacteriology.

[30] O'Toole G, Kaplan HB, Kolter R (2000). Biofilm formation as Microbial development. Annu Rev Microbiol.

[31] Whitfield C, Heinrichs DE, Yethon JA, Amor KL, Monteiro MA, Perry MB (1999). Assembly of the R1-type core oligosaccharide of Escherichia coli lipopolysaccharide. J Endotoxin Res.

[32] Nakao R, Ramstedt M, Wai SN, Uhlin BE (2012). Enhanced biofilm formation by *Escherichia coli* LPS mutants defective in Hep biosynthesis. PloS one.

[33] Genevaux P, MS BPF-DB, DuBow Ms Fau-Oudega B, Oudega B (1999). Identification of Tn10 insertions in the rfaG, rfaP, and galU genes involved in lipopolysaccharide core biosynthesis that affect *Escherichia coli* adhesion. Arch Microbiol.

[34] Stevenson G, Andrianopoulos K, Hobbs M, Reeves PR (1996). Organization of the Escherichia coli K-12 gene cluster responsible for production of the extracellular polysaccharide colanic acid. J Bacteriol.

[35] Van Houdt R, Michiels CW (2005). Role of bacterial cell surface structures in Escherichia coli biofilm formation. Res Microbiol.

[36] Sauer FG, Mulvey MA, Schilling JD, Martinez JJ, Hultgren SJ (2000). Bacterial pili: molecular mechanisms of pathogenesis. Curr Opin Microbiol.

[37] Kaper JB, Nataro JP, Mobley HLT (2004). Pathogenic Escherichia coli. Nat Rev Microbiol.

[38] Wright KJ, Seed PC, Hultgren SJ (2007). Development of intracellular bacterial communities of uropathogenic Escherichia coli depends on type 1 pili. Cell Microbiol.

[39] Reisner A, Maierl M, Jörger M, Krause R, Berger D, Haid A (2014). Type 1 Fimbriae Contribute to Catheter-Associated Urinary Tract Infections Caused by &lt;span class=&quot;named-content genus-species&quot; id=&quot;named-content-1&quot;&gt;*Escherichia coli*&lt;/span&gt. J Bacteriol.

[40] Schilling JD, Mulvey MA, Hultgren SJ (2001). Structure and Function of *Escherichia coli* Type 1 Pili: New Insight into the Pathogenesis of Urinary Tract Infections. J Infect Dis.

[41] Orndorff PE, Falkow S (1984). Organization and expression of genes responsible for type 1 piliation in Escherichia coli. J Bacteriol.

[42] Schwan WR (2011). Regulation of fim genes in uropathogenic Escherichia coli. World J Clin Infect Dis.

[43] Schembri MA, Christiansen G, Klemm P (2001). FimH-mediated autoaggregation of Escherichia coli. Mol Microbiol.

[44] Wurpel DJ, Beatson SA, Totsika M, Petty NK, Schembri MA (2013). Chaperone-usher fimbriae of Escherichia coli. PLoS One.

[45] Korea C-G, Badouraly R, Prevost M-C, Ghigo J-M, Beloin C (2010). Escherichia coli K-12 possesses multiple cryptic but functional chaperone–usher fimbriae with distinct surface specificities. Environ Microbiol.

[46] Barnhart MM, Chapman MR (2006). Curli biogenesis and function. Annu Rev Microbiol.

[47] Mr H, Arnqvist A, Bian Z, Olsén A, Normark S (1995). Expression of two csg operons is required for production of fibronectin- and Congo red-binding curli polymers in Escherichia coli K-12. Mol Microbiol.

[48] Gerstel U, Park C, Römling U (2003). Complex regulation of csgD promoter activity by global regulatory proteins. Mol Microbiol.

[49] Ogasawara H, Yamada K, Kori A, Yamamoto K, Ishihama A (2010). Regulation of the Escherichia coli csgD promoter: interplay between five transcription factors. Microbiology..

[50] Ghigo J-M (2001). Natural conjugative plasmids induce bacterial biofilm development. Nature..

[51] Romeo T, Babitzke P. Global Regulation by CsrA and Its RNA Antagonists. Microbiology spectrum. 2018;6(2). 10.1128/microbiolspec. RWR-0009-2017. 10.1128/microbiolspec.rwr-0009-2017 PMC5868435 29573256

[52] Potts AH, Vakulskas CA, Pannuri A, Yakhnin H, Babitzke P, Romeo T (2017). Global role of the bacterial post-transcriptional regulator CsrA revealed by integrated transcriptomics. Nature communications.

[53] Yakhnin H, Yakhnin AV, Baker CS, Sineva E, Berezin I, Romeo T (2011). Complex regulation of the global regulatory gene csrA: CsrA-mediated translational repression, transcription from five promoters by Eσ^70^ and Eσ(S), and indirect transcriptional activation by CsrA. Mol Microbiol.

[54] Kovach ME, Elzer PH, Steven Hill D, Robertson GT, Farris MA, Roop RM (1995). Four new derivatives of the broad-host-range cloning vector pBBR1MCS, carrying different antibiotic-resistance cassettes. Gene..

[55] Plamont M-A, Billon-Denis E, Maurin S, Gauron C, Pimenta FM, Specht CG (2016). Small fluorescence-activating and absorption-shifting tag for tunable protein imaging in vivo. Proc Natl Acad Sci.

[56] Miller WG, Leveau JHJ, Lindow SE (2000). Improved gfp and inaZ broad-host-range promoter-probe vectors. Mol Plant-Microbe Interact.

[57] Siguier P, Gourbeyre E, Chandler M (2014). Bacterial insertion sequences: their genomic impact and diversity. FEMS Microbiol Rev.

[58] Glansdorff N, Charlier D, Zafarullah M (1981). Activation of gene expression by IS2 and IS3. Cold Spring Harb Symp Quant Biol.

[59] Imamovic L, Misiakou M-A, van der Helm E, Panagiotou G, Muniesa M, Sommer MOA (2018). Complete genome sequence of Escherichia coli strain WG5. Genome announcements.

[60] Grabow WO, Coubrough P (1986). Practical direct plaque assay for coliphages in 100-ml samples of drinking water. Appl Environ Microbiol.

[61] Barrick JE, Yu DS, Yoon SH, Jeong H, Oh TK, Schneider D (2009). Genome evolution and adaptation in a long-term experiment with Escherichia coli. Nature..

[62] Rowe-Magnus DA, Mazel D (2001). Integrons: natural tools for bacterial genome evolution. Curr Opin Microbiol.

[63] Kazazian HH (2004). Mobile elements: drivers of genome evolution. Science..

[64] Malone M, Goeres DM, Gosbell I, Vickery K, Jensen S, Stoodley P (2017). Approaches to biofilm-associated infections: the need for standardized and relevant biofilm methods for clinical applications. Expert Rev Anti-Infect Ther.

[65] SASAKI I, BERTANI G (1965). Growth abnormalities in Hfr derivatives of Escherichia coli strain c. Microbiology..

[66] Chung CT, Niemela SL, Miller RH (1989). One-step preparation of competent Escherichia coli: transformation and storage of bacterial cells in the same solution. Proc Natl Acad Sci U S A.

[67] Tatusova T, DiCuccio M, Badretdin A, Chetvernin V, Nawrocki EP, Zaslavsky L, et al. NCBI prokaryotic genome annotation pipeline. (1362–4962 (Electronic)). 10.1093/nar/gkw569 PMC5001611 27342282

[68] Leung AK, Kwok TP, Wan R, Xiao M, Kwok PY, Yip KY, et al. OMBlast: alignment tool for optical mapping using a seed-and-extend approach. (1367–4811 (Electronic)). 10.1093/bioinformatics/btw620 PMC5409310 28172448

