# Supplementary figures and images for: Genome rearrangements induce biofilm formation in Escherichia coli C – an old model organism with a new application in biofilm research
Source: BMC Genomics. 2019 Oct 22;20:767. doi: 10.1186/s12864-019-6165-4 (PMC6805351; doi:10.1186/s12864-019-6165-4)

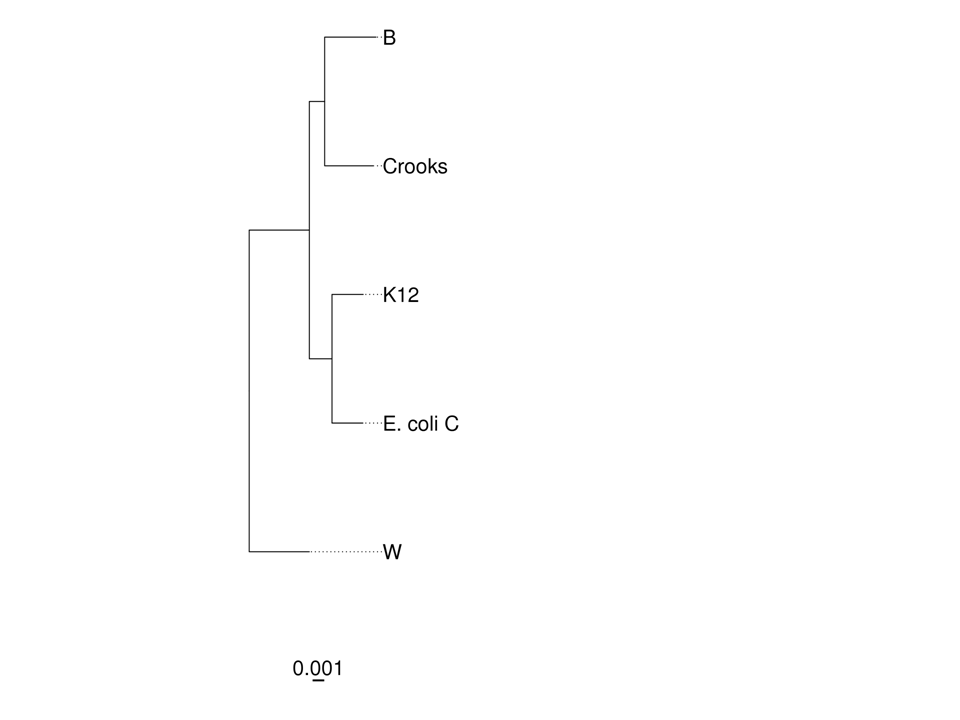

Supplement: Supplementary file 1 — Additional file 1: Figure S1. Maximal likelihood tree based on gene homology within five E. coli strains. [file 12864_2019_6165_MOESM1_ESM.tif]

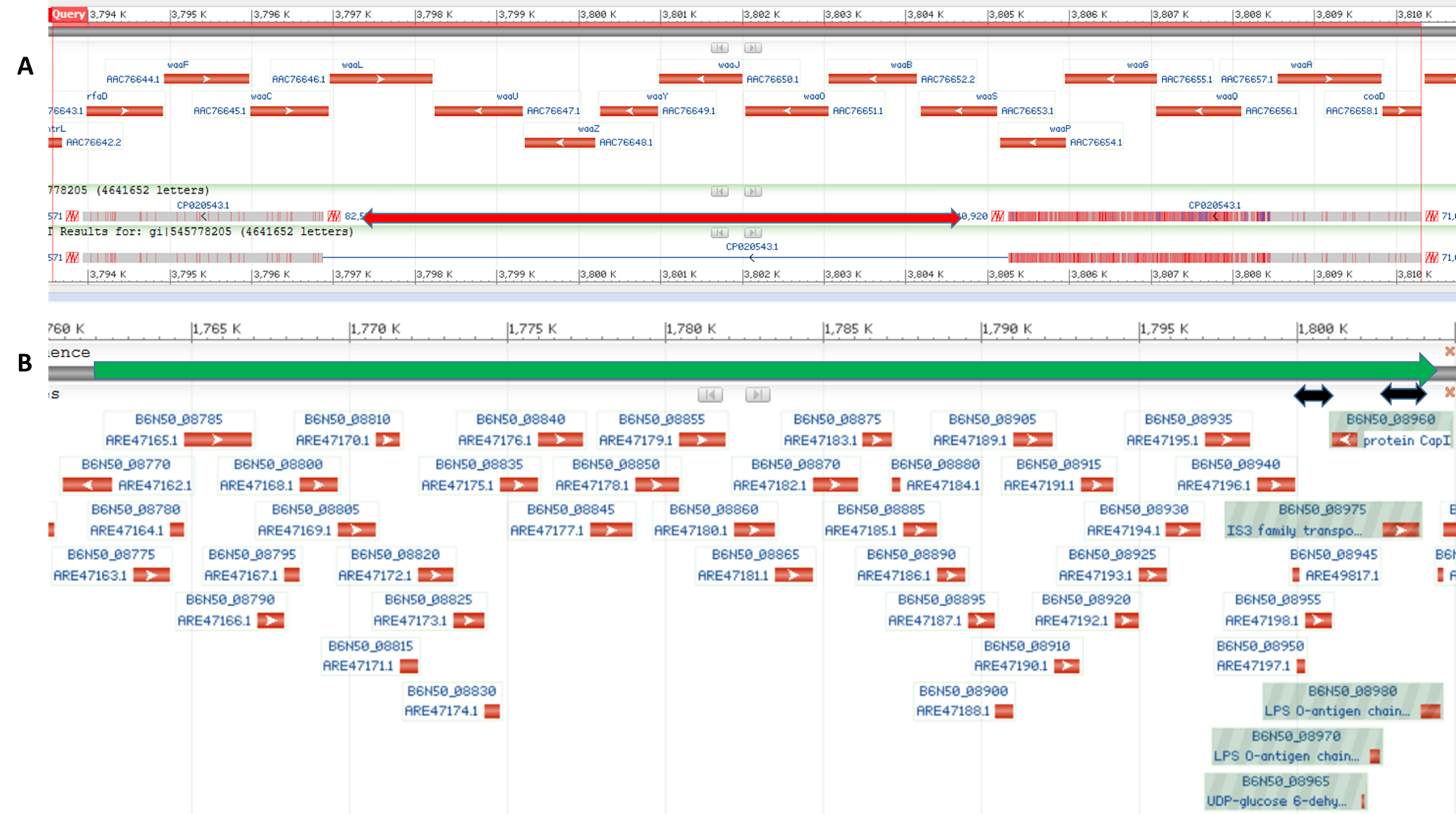

Supplement: Supplementary file 2 — Additional file 2: Figure S2. Genome view of (A) K12 LPS (waa) regions BLAST results with E. coli C genome and (B) colonic acid (wca) region in E. coli C. Red double-headed arrow shows deleted region in strain C. Green arrow indicates a long operon like stretch of 35 genes with IS3 insertions in wzzB and B6N50_08940 genes (black double-headed arrows). [file 12864_2019_6165_MOESM2_ESM.tif]

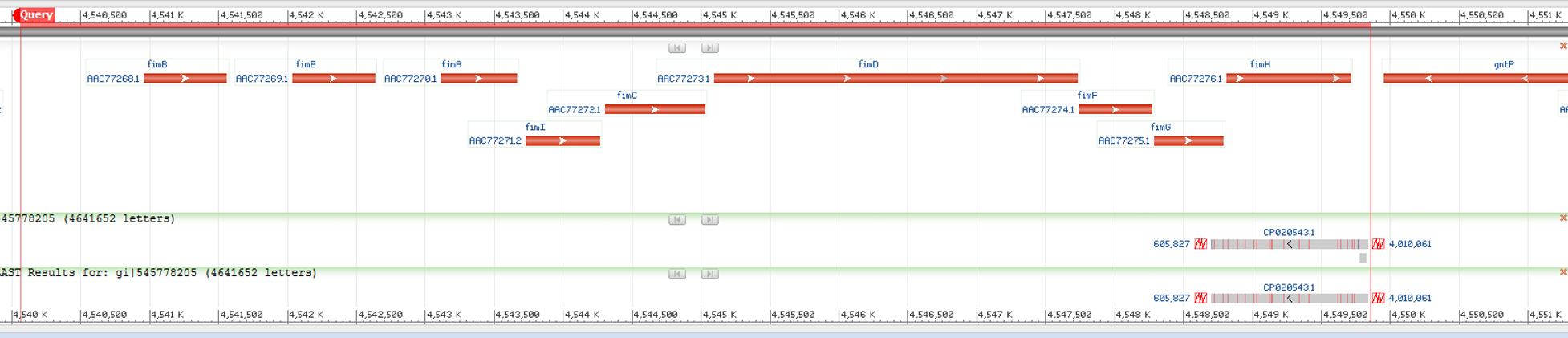

Supplement: Supplementary file 3 — Additional file 3: Figure S3. Genome view of K12 fim region BLAST results with E. coli C genome. Red double-headed arrow shows deleted region in strain C. [file 12864_2019_6165_MOESM3_ESM.tif]

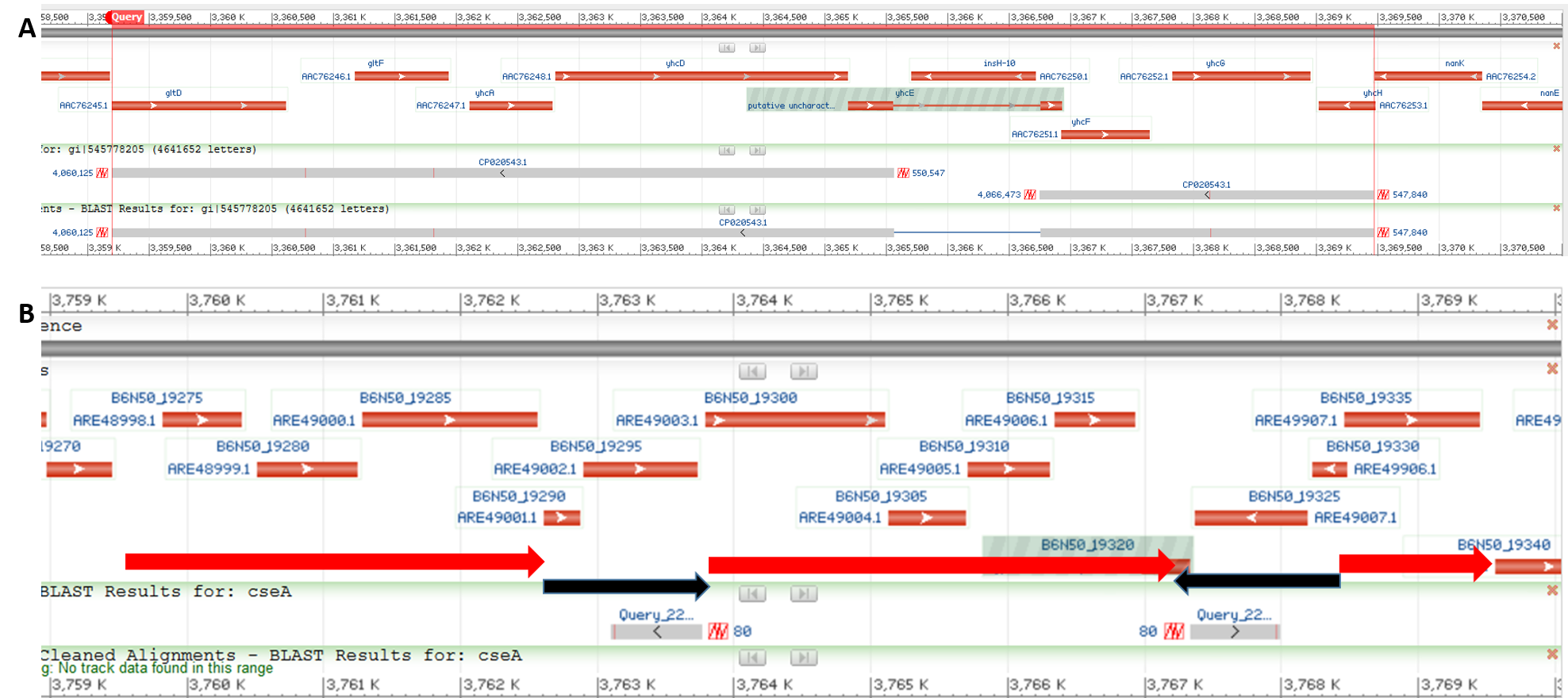

Supplement: Supplementary file 4 — Additional file 4: Figure S4. Genome view of K12 yhc region BLAST results with E. coli C genome (A) and the E. coli C yad region with two IS insertions (black arrows). Deletion of IS5 in E. coli C yhcE gene is highlighted. [file 12864_2019_6165_MOESM4_ESM.tif]

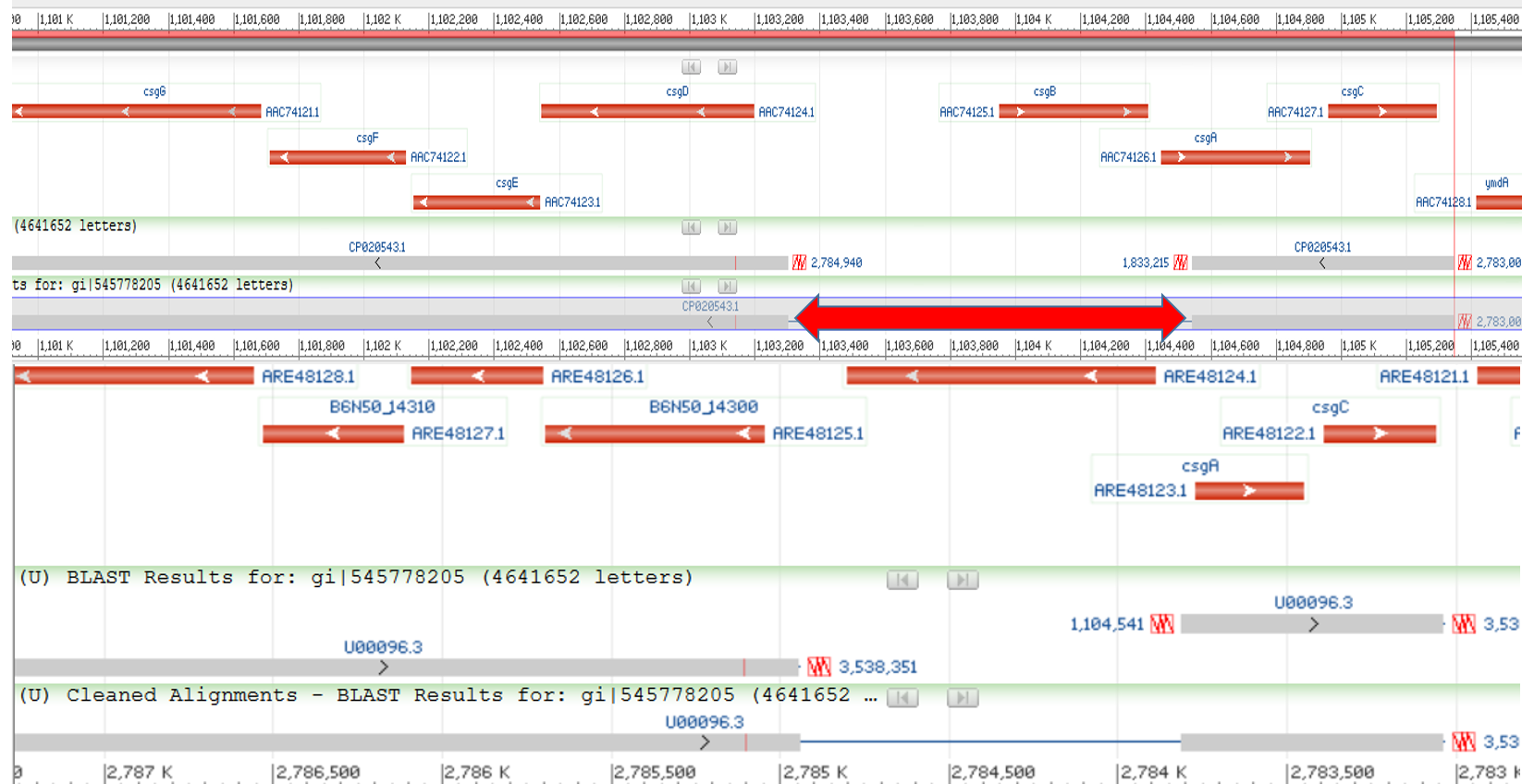

Supplement: Supplementary file 5 — Additional file 5: Figure S5. Genome view of K12 (upper) and C strain (lower) csg region BLAST results with E. coli C genome. Red double-headed arrow shows region replaced by IS5 in strain C. [file 12864_2019_6165_MOESM5_ESM.tif]

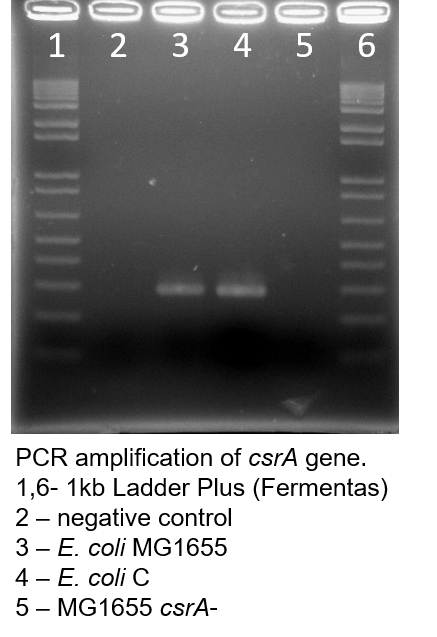

Supplement: Supplementary file 6 — Additional file 6: Figure S6. PCR amplification of the csrA gene from E. coli C and K12 strains. [file 12864_2019_6165_MOESM6_ESM.tif]

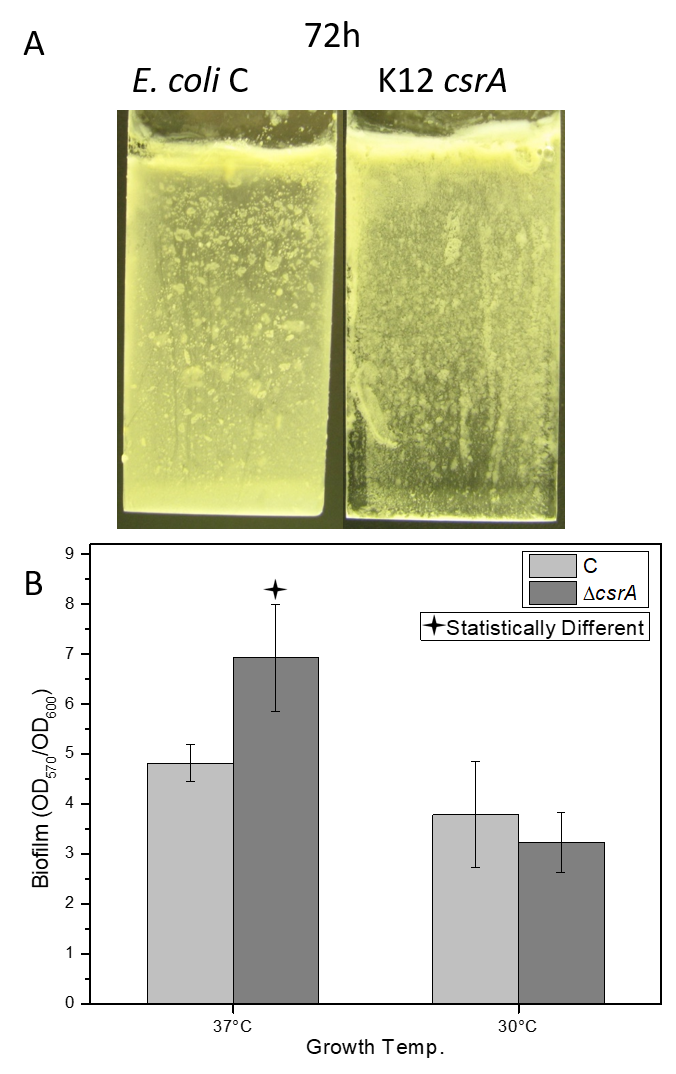

Supplement: Supplementary file 7 — Additional file 7: Figure S7. Biofilm formation by E. coli C and K12 csrA mutant strains on (A) microscope slides (LB medium- 72 h) and (B) 96-well plates (LB Miller broth 37 °C; 24 h). [file 12864_2019_6165_MOESM7_ESM.tif]

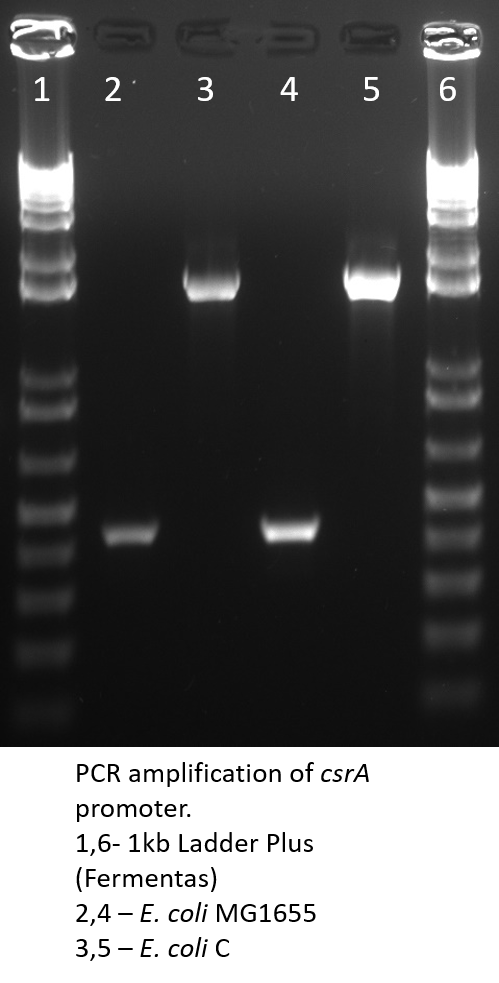

Supplement: Supplementary file 8 — Additional file 8: Figure S8. PCR amplification of the alaS-csrA intergenic region from E. coli C and K12 strains. [file 12864_2019_6165_MOESM8_ESM.tif]

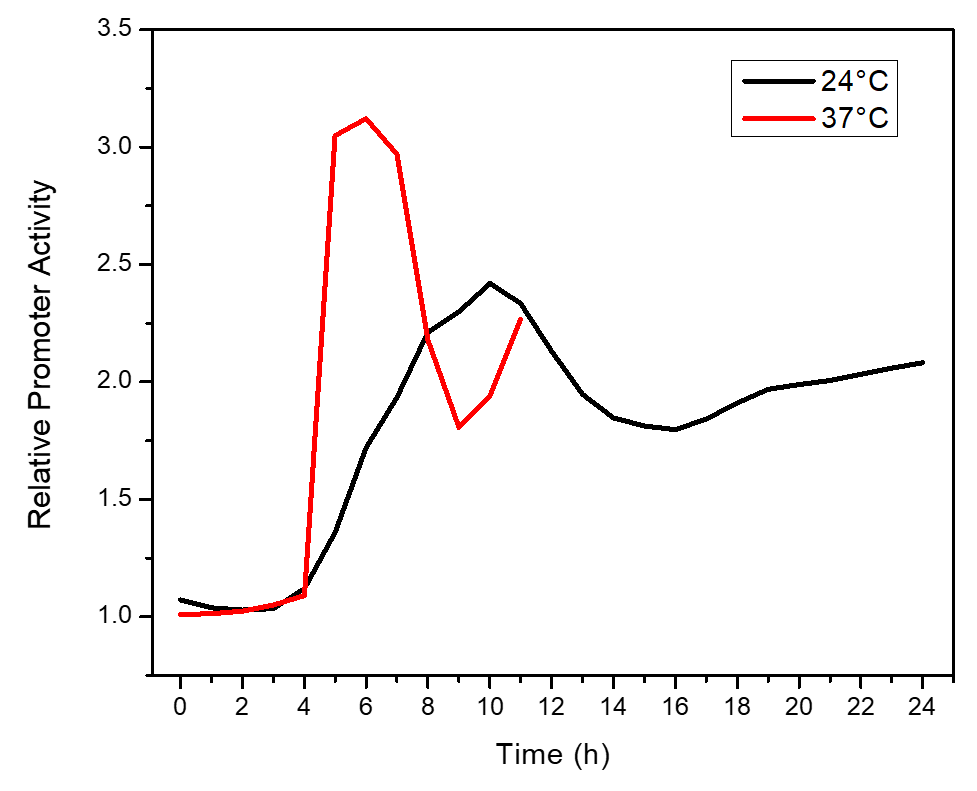

Supplement: Supplementary file 9 — Additional file 9: Figure S9. Differences in relative pcsrA promoter activity between E. coli C and K12 strains grown in LB Miller broth at 24 °C and 37 °C (250 rpm). Cell densities (OD600) and fluorescence (480 nm Ex./520 nm Em.) were measured over the time course to show the relative promoter activity in each strain and condition. The graph represents the ratios between these activities in E. coli C and K12 strains at the specific time points. [file 12864_2019_6165_MOESM9_ESM.tif]

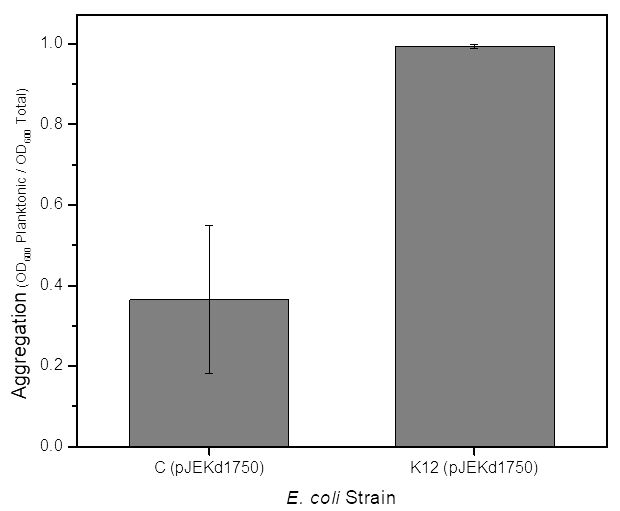

Supplement: Supplementary file 10 — Additional file 10: Figure S10. Cell aggregation of E. coli C and K12 carrying the pJEKd1750 plasmid in overnight culture grown at 37 °C in LB Miller broth on shaker at 250 rpm. Ratio of planktonic cells to total cells measured as OD600. [file 12864_2019_6165_MOESM10_ESM.jpg]
